# Attention network for predicting T-cell receptor–peptide binding can associate attention with interpretable protein structural properties

**DOI:** 10.3389/fbinf.2023.1274599

**Published:** 2023-12-18

**Authors:** Kyohei Koyama, Kosuke Hashimoto, Chioko Nagao, Kenji Mizuguchi

**Affiliations:** ^1^ Laboratory for Computational Biology, Institute for Protein Research, Osaka University, Osaka, Japan; ^2^ National Institutes of Biomedical Innovation, Health and Nutrition, Osaka, Japan; ^3^ Graduate School of Frontier Biosciences, Osaka University, Osaka, Japan

**Keywords:** T-cell receptor, attention networks, transformer, protein structure, peptide, binding prediction, hydrogen bonds

## Abstract

Understanding how a T-cell receptor (TCR) recognizes its specific ligand peptide is crucial for gaining an insight into biological functions and disease mechanisms. Despite its importance, experimentally determining TCR–peptide–major histocompatibility complex (TCR–pMHC) interactions is expensive and time-consuming. To address this challenge, computational methods have been proposed, but they are typically evaluated by internal retrospective validation only, and few researchers have incorporated and tested an attention layer from language models into structural information. Therefore, in this study, we developed a machine learning model based on a modified version of Transformer, a source–target attention neural network, to predict the TCR–pMHC interaction solely from the amino acid sequences of the TCR complementarity-determining region (CDR) 3 and the peptide. This model achieved competitive performance on a benchmark dataset of the TCR–pMHC interaction, as well as on a truly new external dataset. Additionally, by analyzing the results of binding predictions, we associated the neural network weights with protein structural properties. By classifying the residues into large- and small-attention groups, we identified statistically significant properties associated with the largely attended residues such as hydrogen bonds within CDR3. The dataset that we created and the ability of our model to provide an interpretable prediction of TCR–peptide binding should increase our knowledge about molecular recognition and pave the way for designing new therapeutics.

## Introduction

The T-cell receptor (TCR) serves as an antigen receptor, primarily composed of alpha (TCR*α*) and beta (TCR*β*) chains. It has a remarkable sequence diversity in its complementarity-determining regions (CDRs), similar to the B-cell receptor, antibody. The TCR CDR3, found in both *α*- and *β*-chains (CDR3*α* and CDR3*β*, respectively), is the most diverse and vital for recognizing antigenic peptides presented by the major histocompatibility complex (MHC) molecule. The molecule recognized is called the peptide–major histocompatibility complex (pMHC). Given the immense sequence diversity produced through somatic recombination, the potential responses of TCR with different peptides are enormous. Therefore, predicting the TCR–pMHC interaction, primarily involving CDR3–peptide binding, is of great importance. This prediction could significantly impact our understanding of biological functions and disease mechanisms, and guide potential disease recovery pathways.

In response to this, numerous machine learning methods have been developed for the TCR–pMHC prediction (Dash et al., 2017; Gowthaman and Pierce, 2019; Springer et al., 2020; 2021; Lu et al., 2021a; Montemurro et al., 2021; Gao et al., 2023). Some studies in the bioinformatics field were in line with models using the source–target attention (Chen et al., 2020; Honda et al., 2020; Koyama et al., 2020; Weber et al., 2021), and current research studies attempt to apply the attention models to the TCR–pMHC prediction (Xu et al., 2022; 2021; Sidhom et al., 2021; Wu et al., 2021). Notably, when performing predictions of computational models based on cellular assay data regarding the recognition of pMHC by TCRs, the term “TCR–pMHC interactions” is appropriate, despite the absence of MHC or the non-CDR3 TCR sequence in the computational model inputs.

The Transformer (Vaswani et al., 2017) and BERT (Devlin et al., 2018) models, known for their impressive results and interpretability (Voita et al., 2019; Rogers et al., 2020; Hao et al., 2021), have demonstrated the advantages of the cross-attention mechanism in source–target multi-input tasks such as machine translation or image–text classification (Lee et al., 2018; Gheini et al., 2021; Parthasarathy and Sundaram, 2021). Furthermore, during the training process, employing the cross-attention mechanism on two separate sequences is less computationally intensive than applying a self-attention model to concatenated sequences. This is because the computational complexity of the Transformer attention mechanism scales quadratically with the length of the input sequence. Despite the wide application of the Transformer, a comprehensive analysis of interpretability based on the multi-input TCR–pMHC protein complex is yet to be provided. Few studies have attempted to provide the source–target attention model of Transformer at the level of individual residues in CDR3*αβ* or the peptide and analyze structural information such as hydrogen bonds.

For instance, models such as NetTCR-2.0 (Montemurro et al., 2021) and ERGO-II (Springer et al., 2021), despite demonstrating impressive predictive capabilities, are based on convolutional or recurrent neural network frameworks. The PanPep model (Gao et al., 2023), while using an attention mechanism, focuses solely on CDR3*β*. This model provides no information about the structurally important residues on the alpha chains, and it does not account for interaction factors related to hydrogen bonds. The TCR–BERT (Wu et al., 2021) model uses both the alpha and beta chains. However, it is trained without the peptides and does not map the attention on residues for structural analysis. The model proposed by AttnTAP (Xu et al., 2022) utilizes attention, but it does not directly use Transformer attention on both sides of the TCR and peptide. It does not incorporate the alpha chain either. DLpTCR (Xu et al., 2021), another model in this field, employs ResNet attention; however, it refrains from using the Transformer attention.

Unlike existing research, in essence, our model intends to develop a computational method that can incorporate CDR3*α*, CDR3*β*, and a peptide, and conduct a residue-wise structural analysis, leveraging a Transformer-based attention mechanism on sequences. We hypothesize that an attention-based neural network can accurately predict the TCR–pMHC interaction and provide interpretable biological insights into the TCR function and CDR3–peptide binding. To achieve this purpose, we propose a model, the cross-TCR-interpreter, which uses a cross-attention mechanism for predicting the TCR–pMHC interaction, the binding between CDR3 regions of both the *α* and *β* chains, and a peptide.

Our model achieved competitive performance on the benchmark. Furthermore, by performing statistical tests on the attention values over the complex structures, we successfully identified statistically significant structural properties of largely attended residues such as hydrogen bonds and residue distance. We also discuss the limitations of generalizability on unseen data, an issue not unique to our model but evident in other models as well. Our approach, leveraging the source–target attention neural network of Transformer, highlighted the capacity for a deeper understanding and analysis of protein interactions.

## Materials and methods

### Model

**FIGURE 1:**
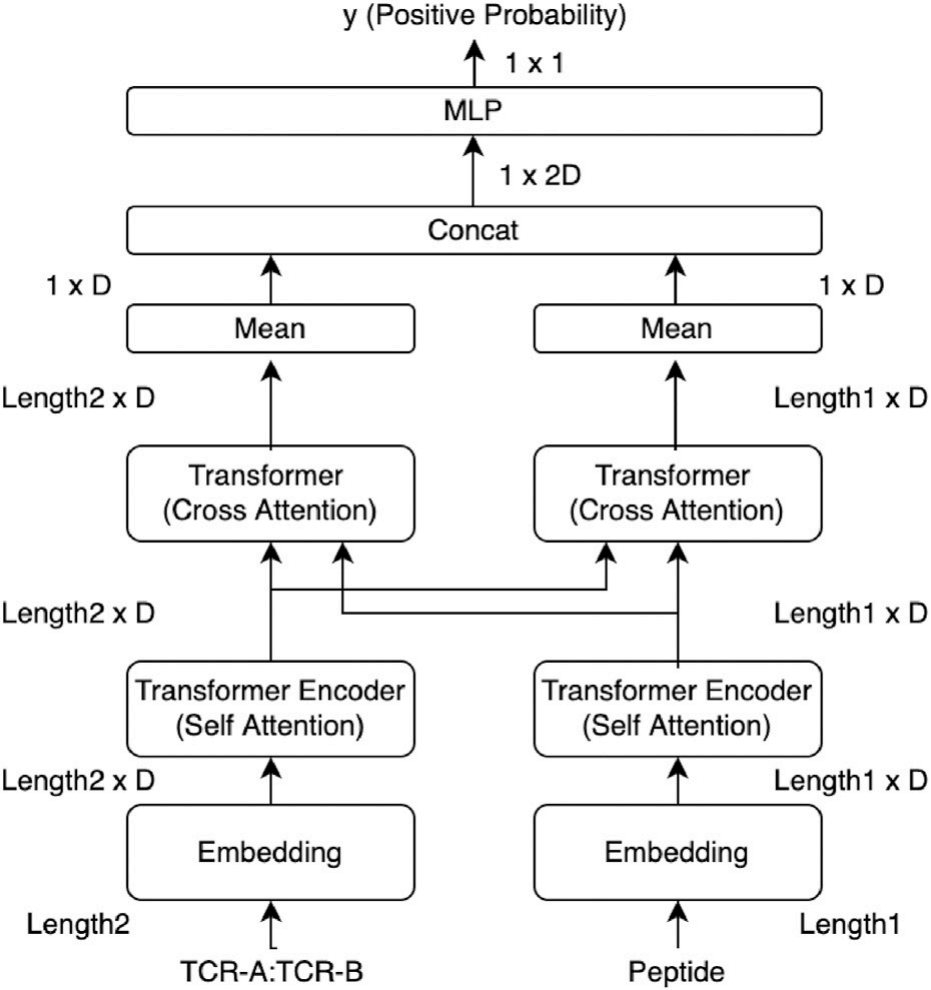
Overview of our cross-TCR-interpreter model. Data tensor sizes are denoted. The cross-attention layers in the middle of the figure were analyzed using structural data after being trained with sequence data. Each embedding layer takes amino acid sequences as the input.

An overview of the prediction model used in this study is shown in Figure 1. The peptide sequence and the sequences of CDR3*α* and CDR3*β* connected with the connection token (colon “:”) were processed separately in the embedding layer and Transformer, and then, they were input into the cross-attention layer designed for the sequence relationship prediction. The cross-attention was used to create a mutual-only layer, enabling the model to verify the relationship. The outputs of the cross-attention layer were concatenated and averaged over the length direction in the output layer. A multi-layer perceptron (MLP) layer outputs a single prediction as a real value, known as the confidence value, from 0 to 1, whereas a true binding datum is represented as a binary value of 0 or 1. Binary cross entropy (BCE) was used as the loss function, and the model output was evaluated using the ROC AUC score and the average precision score.

The model only takes amino acid sequences of CDR3*α*, CDR3*β*, and the peptide as inputs. We used only CDR3s and not the entire TCR sequences. Any other information such as gene types is not utilized. Leveraging solely sequence information, without incorporating domain-specific human knowledge such as gene or MHC information, should be surely the key part for emulating interpretability, closely resembling the natural phenomena of CDR3 binding. The CDR3 and peptide sequences were represented by 20 amino acid residues. Positional embedding and padding tokens were also added to the sequences. Padding at the end of each sequence was performed to ensure the lengths of each CDR3 sequence aligned with the maximum sequence length in the training data; hence, each CDR3*α* had the same length. This was also performed for CDR3*β* and the peptide. The maximum and minimum lengths for the datasets used in this study are provided in Results, while the sequence length distribution is provided in Supplementary Material.

The cross-attention layer is a modified model of Transformer attention. In particular, it takes two sequences as input values and allows meaningful information to be extracted from the entire information about one sequence based on the entire information about the other sequence, implying that it is beneficial for sequence relationship predictions.

The attention layer is specified by Eq. 1.

$$Attention\left(Q,K,V\right)=Softmax\left(QK^{T}/d\right)V.$$ (1)

In Eq. 1 for the attention layer, *Q, K*, and *V* are the data matrices of sequences, and *d* is the scaling factor. *K* ^*T*^ denotes the transposed matrix of *K*, where the sizes of arrays are *Q*: *L*_1_ × *D*, *K*: *L*_2_ × *D*, and *V*: *L*_2_ × *D*. *D* is the embedding dimension. When *Q* = *K* = *V* and *L*_1_ = *L*_2_, this is a self-attention layer.

In the cross-attention layer, *K* (=*V*) and *Q* represent two different inputs, i.e., a connected sequence of CDR3*α*:CDR3*β* and a peptide, respectively.

In addition, we defined four heads for each side in the cross-attention layer, and those heads were concatenated as in a typical Transformer model. The softmax function defines the weights to *V* when the matrix *Q* is the input, and the weights are allocated so that the sum is 1 over the length direction of *V*. This *Softmax* (*QK* ^*T*^/*d*) is the attrition and is used for the analysis and visualization, suggesting the residue positions that are important within the length of *V*.

By representing the learned hidden values of CDR3s, taken from the output of the cross-attention layer just before concatenation, as *H*_*TCR*_, we have

$$\begin{array}{cc} H_{\mathrm{TCR}} & =Softmax\left(Q_{\mathrm{Peptide}}K_{\mathrm{TCR}}^{T}/d\right)V_{\mathrm{TCR}}\;\\ & Attention_{\mathrm{TCR-given-peptide}}\\ & =Softmax\left(Q_{\mathrm{Peptide}}K_{\mathrm{TCR}}^{T}/d\right) \end{array}.$$ (2)

The same was done for the peptide side, and we obtained *H*_*Peptide*_. *H*_*Peptide*_ and *H*_*TCR*_ were concatenated just before the MLP layer.

Visualization and analysis of the attention layer allow interpretation of the residue interaction across sequences (Figure 4). The cross-attention layer uses peptides as inputs and assigns specific weights to each residue of CDR3s to learn the important sites of CDR3s and vice versa. This enabled us to analyze each side of the two areas of attention separately.

All hyperparameters of the model are tuned with the Optuna package (Akiba et al., 2019) and given in Supplementary Material. Except for hyperparameter tuning, the training was completed with one A100 GPU node at the Osaka University SQUID cluster for approximately 3 h, and the inference was completed with a 2.6 GHz 6-Core Intel Core i7 CPU for approximately 2 h.

### Preparation of training and test datasets of sequences

For the TCR–pMHC interaction, especially CDR3s and peptide-binding datasets, we took the repository of ERGO-II (Springer et al., 2021), which contains McPAS (Tickotsky et al., 2017) and VDJdb (Shugay et al., 2018). We also independently downloaded and created the newer version of VDJdb and COVID-19 datasets (Lu et al., 2021b). The sequence datasets we created are as follows:

• **Benchmark datasets McPAS and VDJdb-without10x (training and test)**: The two primary benchmark datasets, McPAS and the VDJdb, were derived from the ERGO-II repository. Specifically, the VDJdb dataset excluded the 10x genomics data (referred to as VDJdb-without10x). These datasets had both training and test sets and contained both positive and negative interactions of TCR–pMHC.
• **Combined data dataset (training)**: For a more comprehensive model training, we also utilized an extended dataset, referred to as the “combined data.” This dataset concatenated the McPAS dataset, VDJdb-without10x, and VDJdb with the 10x genomics data (VDJdb-with10x). This dataset was used for training the model to evaluate the following recent data test set and the COVID-19 dataset.
• **Recent data test set (test)**: To assess the effectiveness of the combined data-trained model in handling new, unseen data, we tested our model on a recent test set from VDJdb downloaded in 2023. The negative TCR–pMHC interactions were added by randomly choosing the CDR3s and peptides as the data contain only positive TCR–pMHC interactions. This evaluates whether a model performs on the most up-to-date data, highlighting its predictive capability for new TCR–pMHC interactions.
• **COVID-19 dataset (test)**: Lastly, to provide a stringent assessment of our combined data-trained model, we created a dataset derived from the study on COVID-19 (Lu et al., 2021b). This dataset is recognized as one of the most challenging for models trained on the combined data dataset.

As this study involves a binary classification problem, negative label data were needed to train the model. However, as most of the TCR and peptide response data are positively labeled, this study followed the same configuration and data on the existing ERGO-II report that generated random CDR3–peptide pairs and assigned negative labels to adjust the positive-to-negative ratio. The size of the negative data was five times larger than that of the positive data. Therefore, each data record to train the model is a tuple of CDR3*α*, CDR3*β*, and a peptide that has a binary label of interaction. When either the CDR3*α* or CDR3*β* sequence was missing for a binary interaction label, the data record of pairs and the label were removed and not used for training.

To ensure no overlapping pairs, we meticulously eliminated the CDR3*α*, CDR3*β*, and peptide pairs from the test set that were present in the training set. However, duplicated pairs of CDR3*α*, CDR3*β*, or individual CDR3 or peptides may still exist that appear in both training and test sets because the same TCR is present in both the test and training datasets and may be paired with other different multiple peptides. The proportion of such duplicates for McPAS and VDJdb is described in the Results section.

### Benchmark dataset and experiment

The validity of the cross-attention model was confirmed by comparing the test scores on the benchmark data using McPAS and VDJdb without 10x Genomics data (VDJdb-without10x). The benchmark models included ERGO-II (Springer et al., 2021) and NetTCR-2.0 (Montemurro et al., 2021), which use both CDR3*α* and CDR3*β*. The only CDR3*β* chain TCR–pMHC prediction models such as NetTCR-2.0 (Montemurro et al., 2021), PanPep (Gao et al., 2023), AttnTAP (Xu et al., 2022), and DLpTCR (Xu et al., 2021) were also compared. In these data, the binary labels were assigned to CDR3*β* and peptide pairs. We evaluated our model performance not only by using the whole test set but also by using the per-peptide score within the test set. The benchmark datasets in the existing ERGO-II research were developed by incorporating assumed negatives, followed by splitting them into training and test datasets. This approach might create an oversimplified problem as many peptides or CDRs are likely to be shared between the training and test datasets.

The detailed benchmark dataset creation process is as follows:

• Step 1: Download the test and training sets of ERGO-II, and remove data records that do not have either one of CDR3*α*, CDR3*β*, or peptide were removed.
• Step 2: Remove data records having duplicated pairs from the test set that are shared with the training set.

When training, we minimized the binary cross-entropy for the training set in the benchmark experiments. If the binary cross-entropy did not improve within 10 updates, we stopped the training. Subsequently, we adopted the weights that provided the minimum value of the binary cross-entropy as the best model.

### The combined data dataset and the recent data test set

After confirmation of the model’s performance, we trained the model again with the whole dataset (i.e., the “combined data” dataset) that included McPAS, VDJdb-without10x, and VDJdb-with10x. Our primary objective with the combined data dataset approach was to uncover meaningful relationships and model the binding nature of the TCR–pMHC interactions, potentially leading to a meaningful interpretation. By using this combined data-trained model, we expected to acquire the learned relationship between the two sequences within the attention layer. The combined data dataset included a 10x dataset (10x Genomics, 2019) that was omitted in the benchmark experiments. By using all the data, we attempted to incorporate the maximum possible information related to binding into the model and herein to analyze the attention weights in the trained model. For the purpose of this model, we designated the test set to comprise the most recent data from VDJdb (i.e., the “recent data” test set), specifically the data downloaded between 2022 and 2023. In contrast, the training set included data downloaded from VDJdb prior to 2022 and McPAS data. After downloading the data, we added five times more negative data records to the downloaded recent data test set. These negative pairs of CDR3s and peptides are sampled only from the recent data test set, not from the combined data dataset. This recent data test set can resemble a realistic situation where we use the model with prospective validation, evaluating the model non-retrospectively.

The detailed combined data dataset and the recent data test set creation processes are as follows:

• Step 1: Download the McPAS, VDJdb-without10x, and VDJdb-with10x data on ERGO-II. Concatenate and remove data records that do not have either one of CDR3*α*, CDR3*β*, or peptide.
• Step 2: Remove duplicated pairs inside the dataset (the combined data dataset).
• Step 3: Download VDJdb in June 2023, and create the pairs of CDR3*α*, CDR3*β*, and the peptide (the recent data test set).
• Step 4: Remove data records having duplicated pairs from the recent data test set that are shared with the training set.
• Step 5: Add five times more negative data records to the recent data test set.

The difference between the recent test and benchmark datasets lies in the timing of the data split. For the recent data test set, we performed the data split prior to adding assumed negative samples to avoid the issue of the oversimplified problem. To show how diverse the recent data and the combined data dataset were, the sequence–sequence pairwise distance matrix was calculated using Clustal Omega software (Sievers et al., 2011) for sequence space analysis.

### COVID-19 datasets and experiment

Similar to the recent data test set, to evaluate how accurately the combined data-trained model would perform in a realistic situation that has no known peptides, we applied it to prediction tasks of a real-world COVID-19 dataset generated from the COVID-19 study (Lu et al., 2021b). A virtual dataset was created using the TCR pairs and peptides taken from the spike (S) protein. In the original study, the reaction between the peptides and TCRs was evaluated by a reporter cell assay by measuring green fluorescent protein expression in the TCR pathway, and the peptides of the S protein were created with a 15-length residue window of amino acid residues by moving four strides of residues. We adopted the same procedure virtually to create the peptides of the S protein, by creating a 9-length residue window and moving one residue stride, as the median length of peptides in the combined data dataset was 9. There was no peptide overlap between the combined data dataset peptides and the 9-length peptides of the COVID-19 dataset. To demonstrate the diversity of the COVID-19 peptides, we computed the sequence–sequence pairwise distance matrix in the same manner, as we did for the combined data dataset.

### Attended residue analysis with attention values on 3D structures

After training the model on the combined data dataset, we could acquire any attention matrix on arbitrary residues. We argue that it makes sense to analyze the model since we used the correctly predicted data. Our approach was not aimed at cherry-picking but rather at investigating and interpreting significant features discerned by the model.

Dividing the residues into two groups of large and small attention made it possible to analyze the attention values. For each head of CDR3 attention being provided a peptide, we defined the residue indices of large CDR3s as *R*_*large,h*_ in Eq. 3.

$$\begin{array}{c} R_{\mathrm{large,h}}=\left\{t|\underset{p}{\max}a_{t,p}>\bar{a}+\gamma \cdot \sigma \right\}\\ \text{where }h\text{ denotes head}\\ \text{and }a_{t,p}\text{ denotes an attention value}\\ \text{of CDR}3\text{ residue index }t\text{ and peptide residue index }p. \end{array}$$ (3)

$$R_{\mathrm{large,all}}=Concat_{h}\left(R_{\mathrm{large,h}}\right).$$ (4)

Eq. 3 shows the TCR-side attention. Given a head *h* of the cross-attention layer, let *A*_*h*_ be an attention matrix of the TCR side with the element *a*_*t*,*p*_. It should be noted that, by the definition in Eq. 1, *∑*_*t*_ *a*_*t*,*p*_ is a one-dimensional all-one vector, $\left(1_{1},1_{2},\cdot \cdot \cdot ,1_{p},\cdot \cdot \cdot ,1_{P}\right)$ , where *P* is the peptide length. We defined this as TCR-side attention because each *p* assigns the attention to TCRs as a sum of one. max_*p*_ takes the maximum value to the peptide axis. $\bar{a}$ is the mean of the attention values of *A*_*h*_, and *σ* is the standard deviation of *A*_*h*_. *γ* is a factor that defines the large or small definition that is empirically expressed in the Results section. When computing the largely attended peptide residues, we exchanged the notation of *t* and *p*. When computing the not-largely attended residues, we replaced the in-equation operator “larger-than” (“>”) with the “smaller-than” symbol (“<”). Eq. 4 shows the attended residues of the TCR side when all heads are concatenated.

### Protein Data Bank structural data analysis

Defining the largely attended residues, the results were examined using a dataset of TCR–pMHC complex structures taken from the Protein Data Bank (PDB) (Berman et al., 2003). We collected TCR-related structures from PDB search and the SCEptRe server (Mahajan et al., 2019), which gathers the complex structures of TCRs. SCEptRe data used here were downloaded on 2 June 2021. With PDB headers, 65 structures with alpha and beta chains were identified. ANARCI (Dunbar and Deane, 2016) was used to extract the CDR3 portion of the structures. These 65 structures were narrowed down to 55 by setting restrictions on the lengths of the TCRs and peptide sequences. The 55 structures contained eight pairs with identical sequences for CDR3s and peptides, and therefore, a final analysis was performed based on the sequences of 47 structures.

We performed a paired Student’s t-test (also called the dependent *t*-test) to assess the differences between the largely attended and not-largely attended residue groups. The paired *t*-test is a statistical method used to compare the means of the two groups of subjects that are dependent on each other. In this study, the TCR–pMHC structures were used as subjects of the *t*-test. The values of the *t*-test were properties such as the proportion of TCR residues that were hydrogen-bonded to the peptide, whether the residue was engaged in an H-bond or not. We used Biopython (Chapman and Chang, 2000) and LIGPLOT (Wallace et al., 1995) to gather the structural properties.

### Input perturbation

To examine individual cases in greater detail, we employed the input perturbation method, which evaluates the sensitivity of a model to changes in its inputs. This approach complements the broader understanding provided by the paired *t*-test of the group.

The input perturbation method involves substituting amino acid residues at some critical positions with alternative amino acids and observing the resulting changes in both prediction and attention values. By altering the attended residues, we assessed the model responsiveness to these modifications, offering the observation of the changes in predictions and attention values.

## Results

### Study overview and experiment types

We performed three experiments to validate the performance and usefulness of our proposed cross-TCR-interpreter model (Figure 1). In the first experiment, we trained and validated the model using existing benchmark datasets, comparing its performances with those of previously proposed models. In the second experiment, in order to conduct the external prospective validation of the TCR–pMHC interaction, we retrained the model using the combined data dataset and validated it with the COVID-19 dataset (Lu et al., 2021b) and the recent data test set. In the third experiment for explainability, we applied the combined data-trained model to a dataset of the TCR–pMHC of known 3D structures, performing statistical analyses of cross-attention values to detail the CDR3–peptide biochemical binding event. Furthermore, we used the model for the input perturbation analysis to observe the change in attention. Hence, although the model was exclusively trained on sequence data, the interpretation of its predictive modeling was further enhanced using structural data.

### Unique element overlap and record-wise overlap can explain the difficulties of datasets

The key statistics of our sequence datasets are given in Table 1. The training records of benchmark datasets are 23,363 for McPAS and 19,526 for VDJdb-without10x. The records of test sets are 4,729 for McPAS and 4,010 for VDJdb-without10x, with no duplicates between the test and the training data. Additionally, the sequence length for each sequence dataset is given in Table 2.

**TABLE 1:** Dataset statistics. The “Interaction” column means the unique count of pairs of {CDR3*α*, CDR3*β*, peptide}, and CDR3*αβ* denotes the unique count of pairs of {CDR3*α*, CDR3*β*}. The duplication count, the “in duplication” row of the “Unique count” column, means the number of unique data that are shared between training and test sets, i.e., overlapped data count. The “Pos. rate” column denotes the positive ratio in the binary label.

Dataset	Unique count	CDR3*αβ*	Peptide	Interaction	Pos. rate
McPAS	In training	3,181	316	23,363	0.1665
McPAS	In test	833	190	4,729	0.1512
-	In duplication b/w training and test	132	171	0	N/A
VDJdb-without10x	In training	2,902	175	19,526	0.1670
VDJdb-without10x	In test	689	120	4,010	0.1504
-	In duplication b/w training and test	111	111	0	N/A
Combined data dataset (A)	In training	23,299	478	119,046	0.1400
Recent data test set (B)	In test	33,183	838	33,360	0.1667
COVID-19 dataset (C)	In test	1,676	1265	2,120,140	1.887 ⋅ 10^–5^
-	In duplication b/w (A) and (B)	18	44	0	N/A
-	In duplication b/w (A) and (C)	1	0	0	N/A

**TABLE 2:** Sequence length for each dataset. For the median and mean, the data record was counted on each data record basis. The distribution of the length is provided in Supplementary Material.

Dataset	Sequence	Max	Min	Median	Mean
McPAS	CDR3*α*	26	6	13	13.27
	CDR3*β*	21	7	14	13.79
	Peptide	25	8	9	9.761
VDJdb-without10x	CDR3*α*	22	5	13	13.37
	CDR3*β*	21	8	13	13.76
	Peptide	20	8	9	9.462
Combined data dataset	CDR3*α*	26	5	14	13.61
	CDR3*β*	26	7	14	14.37
	Peptide	25	7	9	9.520
COVID-19 dataset	CDR3*α*	20	6	14	13.69
	CDR3*β*	21	10	15	14.60
	Peptide	9	9	9	9.00

Table 1 shows unique counts of CDR3s, peptides, and pairs of records. Specifically, for instance, the McPAS training set consists of 23,363 records involving 3,181 unique CDR3 sequences and 316 unique peptides, with 16.67% being positive. From these unique sequences, 833 CDR3s and 190 peptides also appear in the test dataset, whereas none of the same interaction pairs of the CDR3–peptide appear in the test set. Under ideal circumstances, full observations between these unique CDR3 sequences and unique peptides would have yielded a record count of 1,005,196 (=3181 ⋅ 316). However, due to data limitations in real-world datasets, this situation is not realized. There are very few overlapped duplications on CDR3s and peptides between the combined data dataset and the recent data test set. Furthermore, there are very few duplications of CDR3s and peptides between the combined data dataset and the COVID-19 dataset. This explains the difficulty in predicting the TCR–pMHC interactions in the recent data test set and the COVID-19 dataset.

**FIGURE 2:**
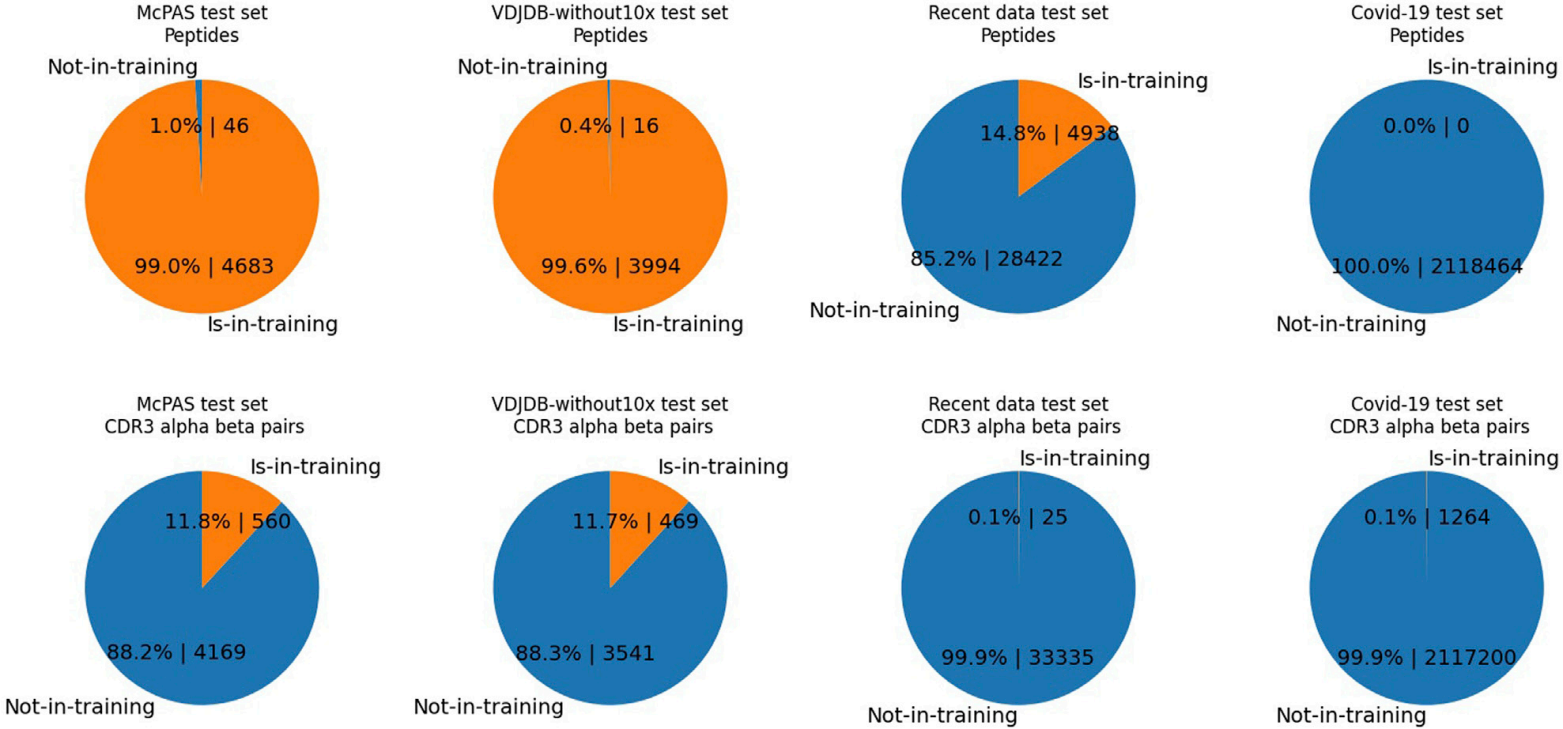
The pie charts illustrate the percentages and counts of data records of the test set comprising either one of the observed elements. Contrary to Table 1, the numbers are the duplication counts of data records, where one side of the peptide or CDRs is shared with the training dataset. In each test set, we display either the duplicated counts of CDR3 pairs or the duplicated counts of peptides. “Is-in-training” indicates that the peptide or CDR3s are present in the training dataset, while “Not-in-training” means the peptide or CDR3s are not found in the training dataset. The test set data record counts of McPAS is 4,729, that of VDJdb-without10x is 4,010, that of the recent data test is 33,360, and that of the COVID-19 test set is 2,120,140. Upper-half row: peptides. Lower-half row: CDR3*αβ*. Each column shows a different dataset; from the left, they are McPAS, VDJdb-without10x test set, the recent data test set, and the COVID-19 test set. For example, the McPAS test set consists of 4,729 records, of which 4,683 records comprise peptides observed in the training set and 46 records comprise brand new peptides. From the CDR3 aspect, 560 records out of 4,729 are composed of unseen CDR3s, whereas 560 records are composed of seen CDRs.

Figure 2 outlines pair-wise duplication within each test dataset, meaning the duplication count of records where one side of the peptide or CDRs is shared with the training dataset. As shown, the test set records of the McPAS and VDJdb-without10x are composed of already observed peptides in the training dataset, while 14.8% of data records of the recent data test set comprise known peptides, and no records of the COVID-19 dataset peptides are observed in the combined data dataset. From both perspectives of peptides and CDRs, the recent data test set and the COVID-19 dataset show records mostly of unseen CDRs or peptides.

For instance, the McPAS test set consists of 4,729 records, of which 4,683 records comprise peptides already observed in the training set and 46 records comprise brand new peptides. However, the recent data test set consists of 33,360 records, and only 4,938 records comprise the peptides observed in the training set.

### The model shows excellent performance for benchmark datasets

To evaluate the performance of our model, we used training and test datasets inspired by those of ERGO-II (Springer et al., 2021). Two benchmark datasets, McPAS and VDJdb-without 10x Genomics data (VDJdb-without10x), were prepared for this experiment. Evaluating the models with the ROC AUC score and the average precision score, our model showed competitive scores against other models for both benchmark datasets in the sequence-feature-only setting models (Tables 3, 4).

**TABLE 3:** Result of the benchmark dataset of McPAS. APS stands for the average precision score.

Model	Features in addition to peptides	ROC AUC	APS
Cross-TCR-interpreter (Ours)	CDR3s of *α* and *β* chains	0.9154	0.6211
NetTCR-2.0	CDR3s of *α*- and *β*-chains	0.9204	0.5808
PanPep	CDR3 sequence of the *β*-chain with biochemical features	0.8374	0.4519
AttnTAP^a^	CDR3 sequence of the *β*-chain	0.840	-
DLpTCR^a^	CDR3 Sequence of the *β*-chain	0.633	-
ERGO-II, LSTM^b^	CDR3s of *α*- and *β*-chains	0.855	-
ERGO-II, LSTM^b^	CDR3s of *α*- and *β*-chains, VJ genes, and MHC type	0.939	-

^a^ The numbers were derived from the AttnTAP paper because we observed that both DLpTCR and AttnTAP achieved only poor scores in our experiments. Hence, to avoid potential misinterpretation due to poor scores, we opted not to display the average precision score in this context. Regarding our experiments of AttnTAP, ROC AUC and APS on McPAS were 0.5934 and 0.3073, respectively. Those of VDJdb-without10x were 0.3951 and 0.1400, respectively. Those of our DLpTCR experiments on McPAS were 0.5346 and 0.1941, respectively, and those of DLpTCR on VDJdb-without10x were 0.5187 and 0.1914, respectively.

^b^ The numbers were derived from the ERGO-II paper.

**TABLE 4:** Result of the benchmark dataset of VDJdb-without10x. APS stands for the average precision score.

Model	Features in addition to peptides	ROC AUC	APS
Cross-TCR-interpreter (Ours)	CDR3s of *α*- and *β*-chains	0.9445	0.7600
NetTCR-2.0	CDR3s of *α*- and *β*-chains	0.9492	0.7262
PanPep	CDR3 sequence of the *β*-chain with biochemical features	0.9009	0.6435
AttnTAP^a^	CDR3 sequence of the *β*-chain	0.894	-
DLpTCR^a^	CDR3 sequence of the *β* chain	0.622	-
ERGO-II, LSTM^b^	CDR3s of *α-* and *β-*chains	0.800	-
ERGO-II, LSTM^b^	CDR3s of *α-* and *β*-chains, VJ genes, and MHC type	0.866	-

^a^ The numbers were derived from the AttnTAP paper because we observed that both DLpTCR and AttnTAP achieved only poor scores in our experiments. Hence, to avoid potential misinterpretation due to poor scores, we opted not to display the average precision score in this context. Regarding our experiments of AttnTAP, ROC AUC and APS on McPAS were 0.5934 and 0.3073, respectively. Those of VDJdb-without10x were 0.3951 and 0.1400, respectively. Those of our DLpTCR experiments on McPAS were 0.5346 and 0.1941, respectively, and those of DLpTCR on VDJdb-without10x were 0.5187 and 0.1914, respectively.

^b^ The numbers were derived from the ERGO-II paper.

**FIGURE 3:**
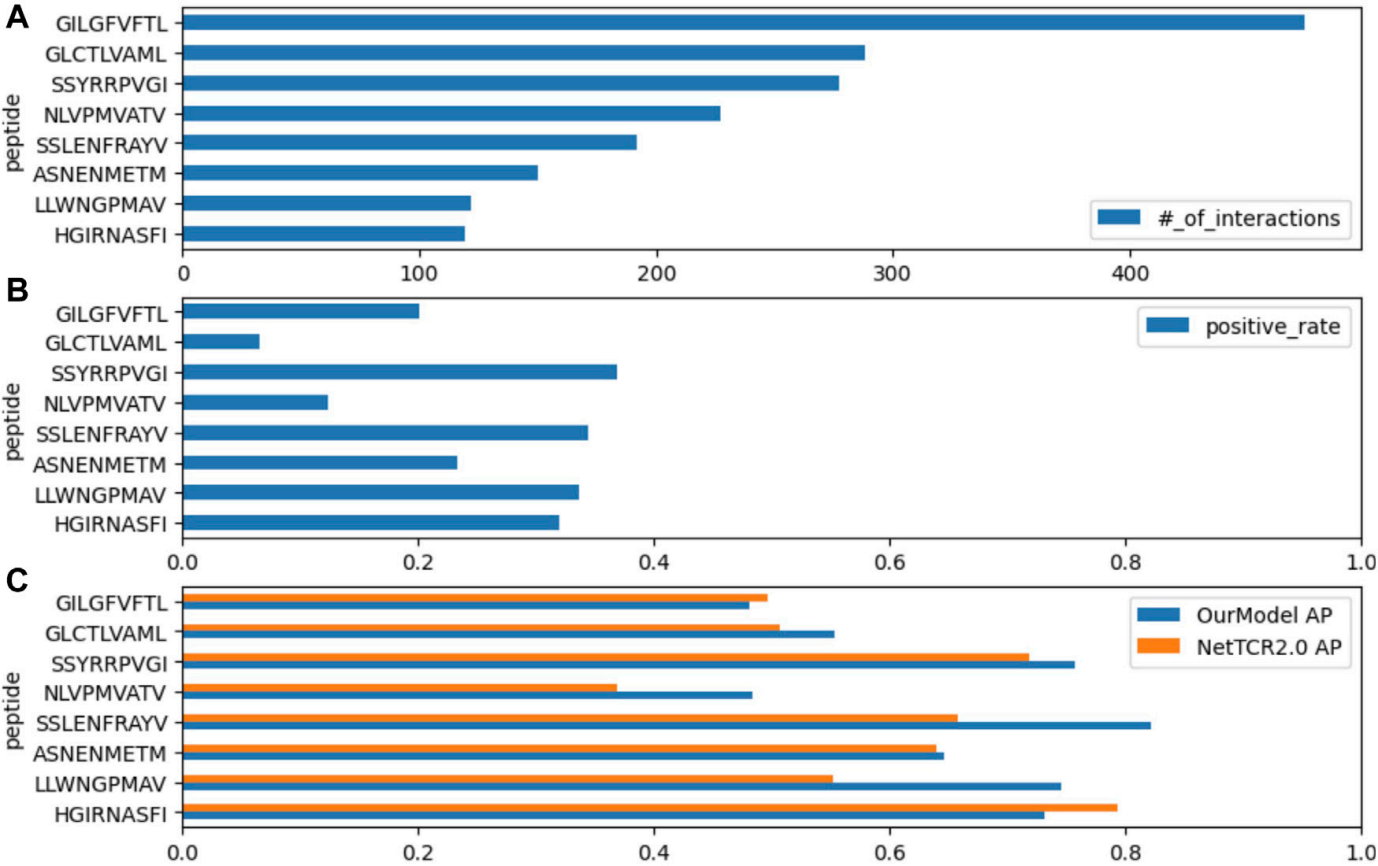
**(A)** Number of records for each peptide, **(B)** positive rate inside the records (the ratio of positively recorded CDR3*αβ*), and **(C)** average precision scores (APSs) in the benchmark data on McPAS.

For detailed performance metrics per-peptide for each test set, we calculated the scores on the top eight frequent peptides shown in Figure 3. Our model shows competitive results over the NetTCR-2.0 (Montemurro et al., 2021) model for the per-peptide performance comparison. We added an analysis of the performance delegation of TCR distance in the Discussion section.

The performance metrics for the best model of ERGO-II were obtained directly from their research paper repository. Their ROC AUC for McPAS and VDJdb was 0.939 and 0.866, respectively. However, they ceased weight updates with the use of the test set, presumably for better use for the code repository users. This prevented us from replicating their top-performing model predictions accurately, thus hindering a fair comparison on the average precision.

### The models exhibit limited performance in the recent data test set

After confirmation of the model performance, we retrained the model with a larger dataset (herein referred to as the “combined data dataset”) consisting of McPAS and the whole VDJdb including the 10x Genomics dataset (10x Genomics, 2019).

Then, we applied our combined data-trained model to recently published data, the recent data test set, evaluating its efficacy in predicting TCR–pMHC interactions in a real-life setting. Our objective with the combined data dataset approach was not necessarily to maximize generalizability but to uncover meaningful relationships and mimic the binding nature of the TCR–pMHC interactions or CDR3–peptide binding. As shown in Table 5, most of the models did not achieve more than 0.9 ROC AUC scores, as in the benchmark, on the pure recent data dataset, as they did in the benchmarks. This result should be explained by the difficulties associated with the number of duplications; it is a difficult task if the records comprise unseen CDRs or unseen peptides.

**TABLE 5:** Result of the recent data test dataset. APS stands for the average precision score.

Model	Dataset	ROC AUC	APS	# of data records	Pos. rate
Cross-TCR-interpreter	Recent data test set	0.5362	0.1855	33,360	0.1667
	Recent data test set of the new peptide subset	0.5085	0.1707	28,422	0.1662
	Recent data test set of the known peptide subset	0.6598	0.3318	4,938	0.1692
	Recent data test set of the new CDR3 subset	0.5355	0.1844	33,335	0.1660
NetTCR-2.0	Recent data test set	0.5274	0.1808	33,360	0.1667
	Recent data test set of the new peptide subset	0.5113	0.1705	28,422	0.1662
	Recent data test set of the known peptide subset	0.6327	0.3008	4,938	0.1692
	Recent data test set of the new CDR3 subset	0.5267	0.1798	33,335	0.1660
PanPep^a^	Recent data test set	0.5337	0.1897	30,221	0.1745
	Recent data test set of the new peptide subset	0.5359	0.1908	25,661	0.1739
	Recent data test set of the known peptide subset	0.5199	0.1852	4,560	0.1779
	Recent data test set of the new CDR3 subset	0.5374	0.1923	29,145	0.1752

The scores for the test set comprising only known CDR3s could not be computed as all the data records are positive.

However, when setting a threshold at 0.5, our model achieves a recall score of 0.56, compared to the NetTCR-2.0 score of 0.44 and PanPep 0.59.

^a^ The datasets employed in our model and NetTCR-2.0 were identical. However, the dataset utilized in PanPep differed due to its exclusive use of a CDR3 beta chain. Consequently, by eliminating duplicates of the beta chain CDR3 from the test set, the total number of data records was reduced from that of our model and NetTCR-2.0.

After the training, the ROC AUC and the average precision score of the training dataset were 0.952 and 0.7952, respectively. Nonetheless, achieving generalizability against the recent data test set posed a significant challenge (Table 5), as evidenced by the ROC AUC and the average precision score of the test set decreasing to 0.5362 and 0.1855, respectively. By restricting the data records of the test set to the known peptides, we did observe relative improvements in the average precision scores, increasing to 0.3318. By restricting the data records of the test set to the new peptides that were not observed in the training dataset, we observed a decrease in the average precision score to 0.1707. Not only does our model demonstrate poor performance but also the NetTCR-2.0 or PanPep models exhibit a similar level of performance deficiency in the recent data test set and its subsets. Regarding the PanPep model in which we used a zero-shot model setting for the unseen peptides and a majority model setting for the known peptides, while it claims to predict CDR3*β*–peptide pairs for unseen peptides, it achieved a slightly better average precision score of 0.1897, outperforming our model by a small margin. Nevertheless, for the data records of the test data subset of the known peptide, it only achieved less than the new peptide setting. These performances are still insufficient to serve as a viable alternative to wet laboratory experiments. Hence, it is clear that predicting CDR3–peptide interactions that contain peptides not represented in the training data remains a considerable challenge.

### Our model does not exhibit satisfactory performance for the COVID-19 dataset

We also applied our combined data-trained model to a recently published COVID-19 dataset (Lu et al., 2021b), evaluating its efficacy in predicting the TCR–pMHC interactions in a real-life setting. As described in the Methods section, peptides from each SARS-CoV-2 protein were created with a 9-length residue window by moving one stride. No peptides of the COVID-19 dataset were found in the combined data dataset. The total number of data records was 2,120,140, of which 2,120,100 were negative data records and only 40 data records were positive. Of the 2,120,140 data records, there were 1,676 unique CDR3 alpha–beta pairs and 1,265 unique peptides (1, 676 ⋅ 1, 265 = 2, 120, 140, as shown in Table 1). Of the 40 positive records, we found 10 unique CDR3*αβ* pairs and 24 unique peptides. Consequently, this means that the remaining 200 records, composed of these specific CDR3s and peptides, are classified as negative records (10 ⋅ 24–40 = 200).

By maximizing the F1-score of this prediction task, the model achieved a precision score of 2.501 ⋅ 10^–5^ and a recall of 0.600. In the confusion matrix, the true positive count was 24, the false negative count was 16, the false positive count was 959,512, and the true negative count was 1,160,588. The ROC AUC score was 0.5461, and the average precision score was 2.032 ⋅ 10^–5^. Given the fact that positive records exist at a rate of 1.887 ⋅ 10^–5^ (=40/2120140), we can claim that the model can detect positive records 1.326 times (=2.501/1.887) better than the random selection, but its specificity was not adequate enough to replace wet laboratory experiments.

### Residues in structural data are categorized based on their level of attention into largely attended and less attended groups

Although perfect generalizability was not achieved, we sought to interpret the model within the 39 complex structures, where the model surely performs well enough to analyze. Using the procedure described in the Methods section, we started with 47 TCR-related structures from the PDB search and the SCEptRe server. Of these 47 structures, our model designated 39 structures as having positive TCR–pMHC interactions, using a threshold of 0.5. A notable observation was that 30 of these 39 structures share sequences with the combined data dataset. We paid special attention to these 39 cases in our analysis of attention layers, on the premise that the accurate interpretability of the model could be safely assumed for these instances. This is similar to a regression analysis examining the effect of some explanatory variables on target variables, and our goal was to identify the important features that the model learns, i.e., the features of the largely attended amino acid residues. Details of these 39 structures are given in Supplementary Material.

The attention values were considered “large” when they exceeded the threshold of MEAN + 5.5 STD on the peptide side and MEAN + 4.5 STD on the TCR side (5.5 and 4.5 are *γ*s in Eq. 3). Approximately 20% of the residues were identified as large on each side, using *γ* as a result of the total sum of the four heads. The thresholds were determined through empirical evaluation, and the residue count generated by changing *γ* is provided in Supplementary Material. The chosen thresholds were found to be effective in differentiating between large and small attention values. It should be noted that the threshold for large attention values varies for each PDB entry or head due to differences in the distribution of attention values.

The analysis was performed separately for each of the four heads in the cross-attention layer (heads 0–3) on both the TCR and the peptide sides, with each head being analyzed separately. The cross-attention layer was defined on a CDR3*αβ* sequence and a peptide sequence, resulting in an attention matrix with a shape determined by the length of the peptide and CDR3*αβ* residues. It was possible for a particular residue to have a large attention value in head 0 but not in the other heads (as observed in Eq. 3).

**FIGURE 4:**
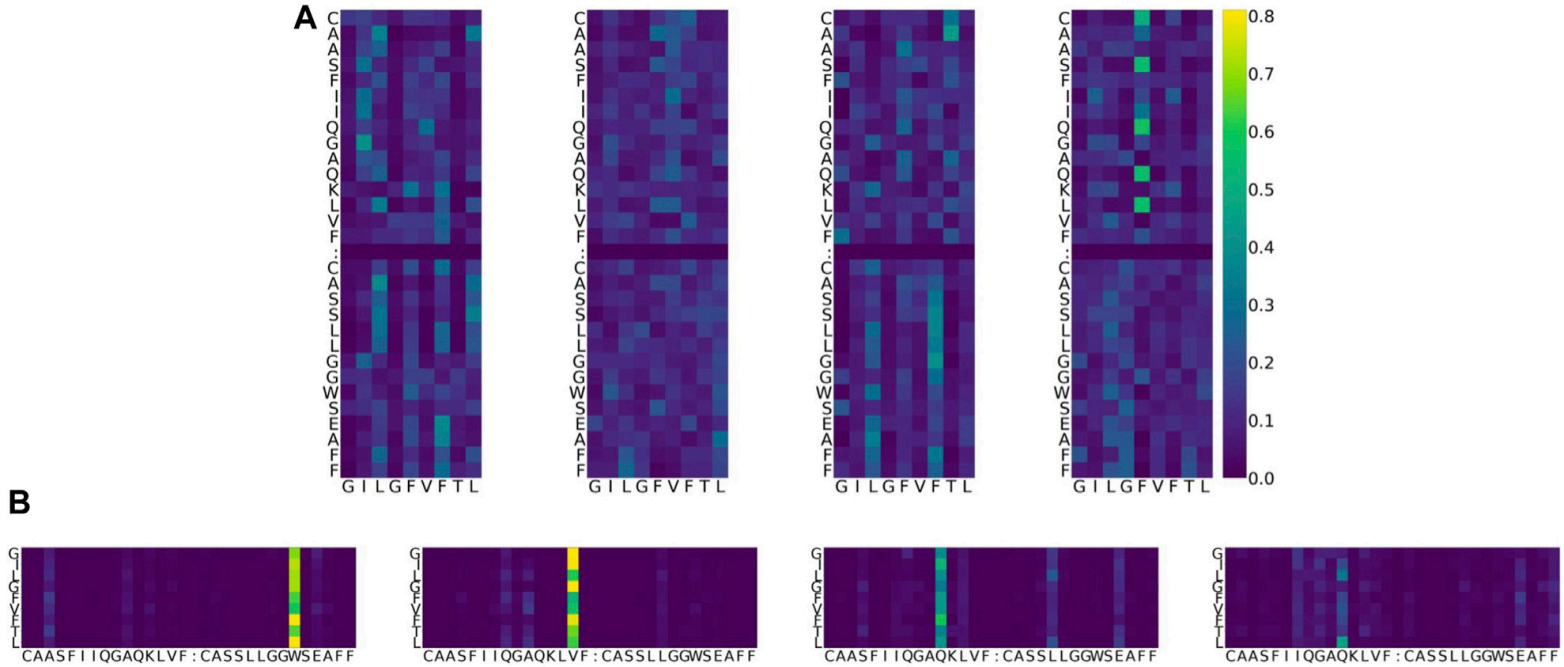
Example of attention value visualization of PDB ID 5TEZ (Yang et al., 2017). **(A)** Upper half: attention values of a peptide so that the sum is 1 over the peptide, given a CDR3 *αβ* pair. The x-axis represents the residue of the peptide, while the y-axis represents the residue of TCRs. Lower half: attention values of a CDR3 pair so that the sum is 1 over the CDR3s, given a peptide. The x-axis is the residue of TCRs, while the y-axis is the residue of peptides. The sum over the x-axis direction is 1 for both images. Four columns denote the heads of the multi-head attention layer. Colors denote the magnitude of the attention value: dark blue represents smaller attention, yellow represents larger attention, and green is in the middle. **(B)** In the lower figure, the cell corresponding to the peptide position *L*_8_ (the last row) and CDR3*β* position *W*_24_ (sixth column from the right) represents the weight of how important the CDR3 *W*_24_ is, given the peptide *L*_8_. It is denoted in bright yellow, which means that the attention value is large, and the two residues might play a potentially biologically important role during predictions. Furthermore, this value is larger than the MEAN + *γ* STD threshold, defined for each PDB ID and each head.

**FIGURE 5:**
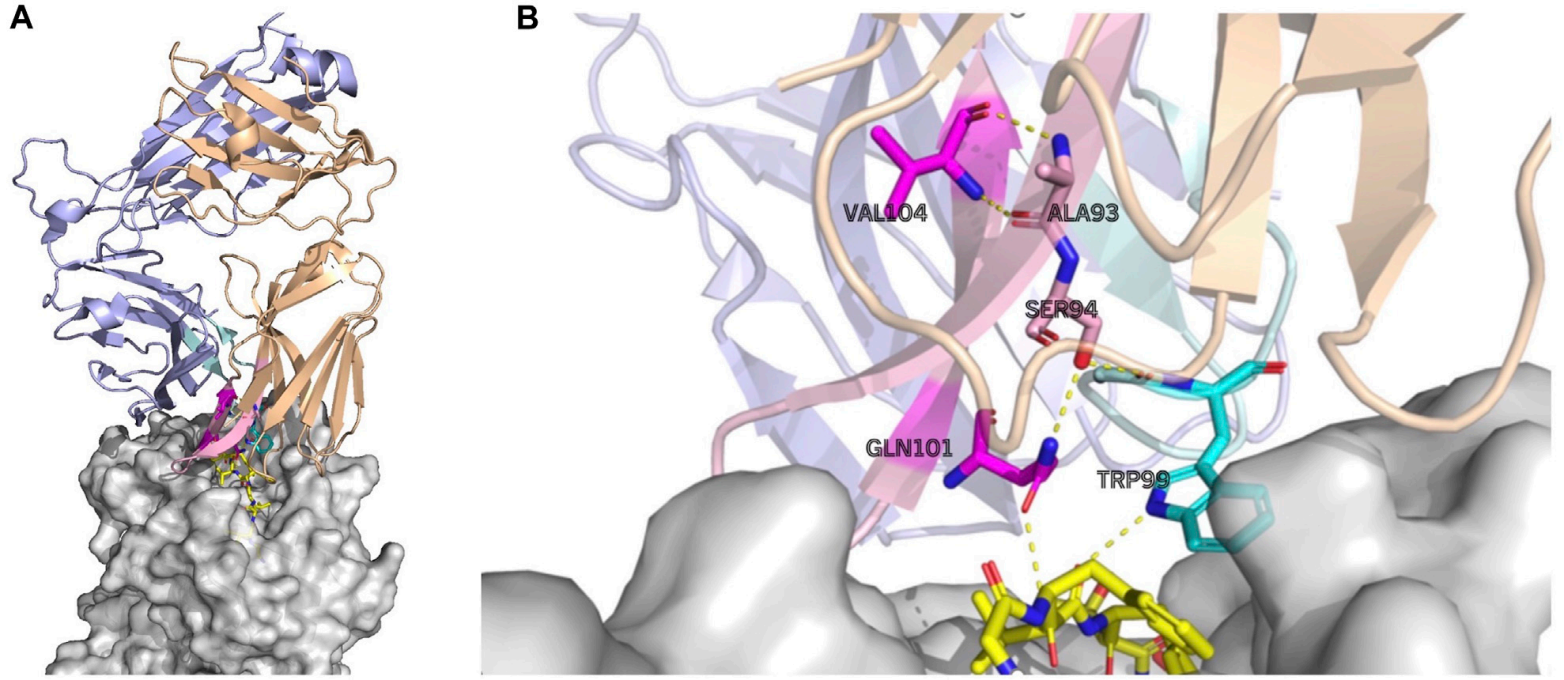
Largely attended residues in the TCR—the influenza virus epitope–HLA complex (PDB ID: 5TEZ)—where the CDR3 sequences of TCR *α* and *β* are CAASFIIQGAQKLVF and CASSLLGGWSEAFF, respectively. The left figure **(A)** shows the overall structure of the complex, and the right figure **(B)** shows the residue interactions of the largely attended residues: VAL104 (the 14th Val(V) of TCR *α*) and GLN101 (the 11th Gln(Q) of TCR *α*) of the TCR *α* chain, and TRP99 (the 9th Trp(W)) of the TCR *β* chain. The TCR *α* chain is wheat, the *β*-chain is light blue, the TCR *α* CDR3 part is light pink, and the *β* CDR3 is pale cyan. The residues with the large attention in CDR3 *α* are denoted in magenta and that in TCR *β* CDR3 is cyan. The MHC is denoted in gray. The residues with large attention and interacting residues are represented by sticks. The yellow dot lines represent the hydrogen bonds. VAL104 makes the two hydrogen bonds bind to TCR *α* ALA93 (the 3rd alanine Ala(A) of TCR *α*) and may contribute to the stabilization of the end of the CDR3 loop conformation. GLN101 is hydrogen-bonded with TCR *α* SER94, and SER94 is hydrogen-bonded to TCR *β*, maintaining the *α* and *β* structures. GLN101 of TCR *α* and TRP99 of *β* have hydrogen bonds with the epitope. PyMOL (Schrödinger and Delano, 2020) is used for visualization.

As an example, the attention values for the TCR–pMHC of PDB entry 5TEZ are shown as eight heatmaps in Figure 4. 5TEZ has a complex structure of MHC class I HLA-A2, influenza A virus, and TCRs (Yang et al., 2017). The corresponding 3D structure of the TCR–pMHC is shown in Figure 5, in which the amino acid sequences of the peptide, CDR3*α*, and CDR3*β* are GILGFVFTL, CAASFIIQGAGKLVF, and CASSLLGGWSEAFF, respectively.

### Statistical analysis shows largely attended residues form H-bonds with CDR3

Using the *γ* factor of 4.5 defined in Eq. 3, we classified the TCR residues into two groups based on their attention values, “large” and “small,” for the cross-attention of the TCR side, given a peptide. To gain insights into the characteristics of each group of residues, we analyzed their structural properties.

To assess differences between the two groups, we performed a paired *t*-test to remove variations arising from individual structural factors. In this study, 39 TCR–pMHC structures were used as subjects, and structural properties associated with large- or small-attention groups were the tested values. The purpose of the paired *t*-test was to examine the null hypothesis that the mean difference between the pairs of measurements is zero. The proportion of a property, the test value (e.g., H-bonded to any peptide residue), is calculated by *P* = *A*_*h*_/*B*_*h*_, where *A*_*h*_ is the number of residues with one or more H-bonds of the specified type within the residues of large attention values and *B*_*h*_ is the number of residues of large attention values, where *h* denotes the head.

The results of the statistical tests are shown in Table 6. Although each head was analyzed equally and separately, they showed different results. The results of all concatenated heads are shown in Table 6. The individual results for each head are given in Supplementary Material.

**TABLE 6:** TCR-side attention analysis. Structural property comparisons between the large- and small-attention residue groups are shown. The *p*-adjusted column shows the adjusted *p*-value by the Benjamini–Hochberg (BH) procedure. The symbol “***” denotes the significant difference based on a false discovery rate (FDR) of 0.05 in the BH procedure; the symbol “**” indicates significance at an FDR value of 0.10; and the symbol “*” indicates an FDR value of 0.15. The numbers in the Large attention or Small attention columns are the average and standard deviation, respectively.

Property	Large attention^a^	Small attention^a^	*p*-value	*p*-adjusted	
H-bonded to any peptide residue	0.0862 ± 0.1368	0.0805 ± 0.0675	0.828	0.9108	
H-bonded to any CDR3 residue	0.4846 ± 0.2216	0.4103 ± 0.1040	0.0478	0.1315	*
H-bonded to any non-CDR3 TCR residue	0.2940 ± 0.1923	0.4672 ± 0.0846	3.88e-05	4.268e-04	***
H-bonded to any TCR residue	0.6845 ± 0.1650	0.7294 ± 0.0880	0.0987	0.2145	
H-bonded to any CDR3 residue of its own chain	0.4643 ± 0.2180	0.3752 ± 0.0922	0.0107	0.05885	**
H-bonded to any TCR residue of its own chain	0.6013 ± 0.1999	0.6561 ± 0.0880	0.117	0.2145	
H-bonded to any TCR residue of the opposite chain	0.1679 ± 0.1714	0.1497 ± 0.0793	0.562	0.7199	
H-bonded to any CDR3 residue of the opposite chain	0.0306 ± 0.0857	0.0672 ± 0.0743	0.0369	0.1315	*
In the edge^b^	0.6434 ± 0.2064	0.5928 ± 0.0570	0.218	0.3426	
Closest distance to the peptide (Å)^c^	8.4072 ± 2.2892	8.4122 ± 0.9592	0.988	0.988	
Number of H-bonds formed^c^	2.0234 ± 0.9370	2.0875 ± 0.6685	0.589	0.7199	

^a^ Mean and standard deviation (for the 39 structures) of the proportion of residues that satisfy the property shown in the first column.

^b^ Four residues from the beginning and four from the end of the CDR.

^c^ In the last two properties, per-residue averages were used instead.

As a TCR sequence for the structural analysis includes both CDR3 and non-CDR3 portions, the H-bond properties were measured by dividing the residues into CDR3 and non-CDR3 portions.

The residues with large attention values had a more significant proportion of having an H-bond with the CDR3 portions inside their own chain. Nonetheless, the proportion of residues that are H-bonded to any TCR residue (i.e., H-bonds within the TCR chains) showed no difference between the large- and small-attention groups.

A natural consequence of those observations is that the largely attended residues are less likely to be H-bonded to the non-CDR3 portions, compared to the residues with small-attention values. The most significant property difference in all concatenated heads occurred in the proportion of H-bonded to any non-CDR3 TCR residue. This means that the largely attended residues are highly likely to avoid the H-bonded to the non-CDR3 TCR part, whereas they are likely to have H-bonds with the CDR3 portions.

To avoid pitfalls associated with multiple *p*-values in the statistical analysis, we executed the Benjamini–Hochberg (BH) procedure and adjusted the *p*-values. Here, the “false discovery rate (FDR) for BH” represents the likelihood of incurring a type I error among all rejected null hypotheses. At an FDR threshold of 0.05, only the “H-bonded to any non-CDR3 TCR residue” hypothesis was rejected, demonstrating the rigor of this threshold. Although many assertions in our study might be substantiated when considering average metrics, they may not attain statistical significance at this level. Meanwhile, modifying FDR to 0.1 led to the rejection of two hypotheses, “H-bonded to any non-CDR3 TCR residue” and “H-bonded to any CDR3 residue of own chain,” which is additionally highlighted by the symbol “**” in Table 6. Further increasing FDR to 0.15 expanded the rejections to four hypotheses, adding “H-bonded to any CDR3 residue of opposite chain” and “H-bonded to any CDR3 residue,” which are designated by “*” in Table 6. Collectively, these statistical evaluations lend support to our hypothesis that attended residues significantly avoid H-bonds with non-CDR3 TCR regions, favoring H-bonds within the CDR3 regions.

In contrast, contrary to expectations, the proportion of largely attended TCR residues to form an H-bond with any peptide residues was not significant in all heads. This highlighted a surprising and counter-intuitive finding in our analysis. We also examined the closest distance from a given TCR residue to any peptide residue; however, no significant difference was observed.

We also performed a similar analysis on the peptide side (Table 7) and observed that amino acid residues with large attention values had smaller distances to the closest TCR residues, a pattern not observed on the TCR side. This poses an interesting structural aspect.

**TABLE 7:** Peptide-side attention analysis. Structural property comparisons between the large- and small-attention residue groups are shown.

Property	Large attention^a^	Small attention^a^	*p*-value	*p*-adjusted
H-bonded to any peptide residue	0.0495 ± 0.1443	0.0659 ± 0.1206	0.458	0.56
H-bonded to any CDR3 residue	0.2050 ± 0.3024	0.1682 ± 0.0982	0.48	0.56
H-bonded to any TCR residue	0.3401 ± 0.3714	0.2372 ± 0.1184	0.151	0.3523
H-bonded to any non-CDR3 TCR residue	0.1712 ± 0.3112	0.1118 ± 0.1283	0.355	0.56
In the edge^b^	0.4459 ± 0.4097	0.5874 ± 0.1232	0.0795	0.3523
Closest distance to the peptide (Å)^c^	4.6398 ± 1.7149	5.1926 ± 1.2647	0.141	0.3523
Number of H-bonds formed^c^	2.1126 ± 1.4959	2.0031 ± 0.9051	0.668	0.668

^a^ Mean and standard deviation (for the 39 structures) of the proportion of residues that satisfy the property shown in the first column.

^b^ Three residues from the beginning and three from the end of the peptide.

^c^ In the last two properties, per-residue averages were used instead.

### Impact of largely attended residues on model behaviors through input perturbation analysis

In this subsection, we delve into the effect of input perturbations on the outcomes of predictions and attention values through modification of the input sequence. This technique was utilized on the training data, PDB ID 5TEZ. Furthermore, we extended this approach to a mutation study (Cole et al., 2013), which was not a part of our training data. The study involved mutating the protein sequence of the CDR3 beta loop of A6-TCR and assessing its binding strength against the TAX peptide, a peptide of the human T-cell leukemia virus type I, on the MHC class I HLA-A2. The sequences and structures, following mutation, were recorded in the PDB under the identifiers, PDB ID 1AO7 (before mutation) and PDB ID 4FTV (after mutation).

For the 5TEZ PDB structure, three residues exhibited large attention values, 11th Gln(Q) of CDR3*α*, 14th Val(V) of CDR3*α*, and 9th Trp(W) of CDR3*β* (Table 8). We assessed how prediction and attention values were affected when these residues were substituted with alternative amino acids. The CDR3*α* sequence of 5TEZ is *CAASFIIQGAQKLVF*, while CDR3*β* is *CASSLLGGWSEAFF*. Notably, 11th Gln(Q), 14th Val(V), and 9th Trp(W) formed H-bonds, but only the 14th Val of the *α*-chain formed two H-bonds with the internal CDR3 chain of the TCR residue.

**TABLE 8:** CDR3 chain analysis for 5TEZ, 1AO7 (before mutation), and 4FTV (after mutation).

5TEZ	*α* chain	*β* chain
AA types	CAASFIIQGAQKLVF	CASSLLGGWSEAFF
Large or small attention	SSSSSSSSSS**L**SS**L**S	SSSSSSSS**L**SSSSS
# of H-bonds	212421222253322	22453423486223
# of H-bonds with self-CDR3	102120200213120	10220300140020
# of H-bonds with the peptide	000000000010000	00000000100000
1AO7	*α* chain	*β* chain
AA types	CAVTTDSWG	CASRPGL**AGGR**P
Large or small attention	SS**L**SSSSS**L**	SSSSSSSSSSSS
# of H-bonds	122325223	124610210212
# of H-bonds with self-CDR3	102013011	002200010012
# of H-bonds with the peptide	000000200	000100100000
4FTV	*α*-chain	*β*-chain
AA types	CAVTTDSWG	CASRPGL**MSAQ**P
Large or small attention	SS**L**SSSSS**L**	SSSSSSSSS**LL**S
# of H-bonds	222428312	224711101112
# of H bonds with self-CDR3	102114011	002200001012
# of H-bonds with the peptide	000000200	000000000000

**FIGURE 6:**
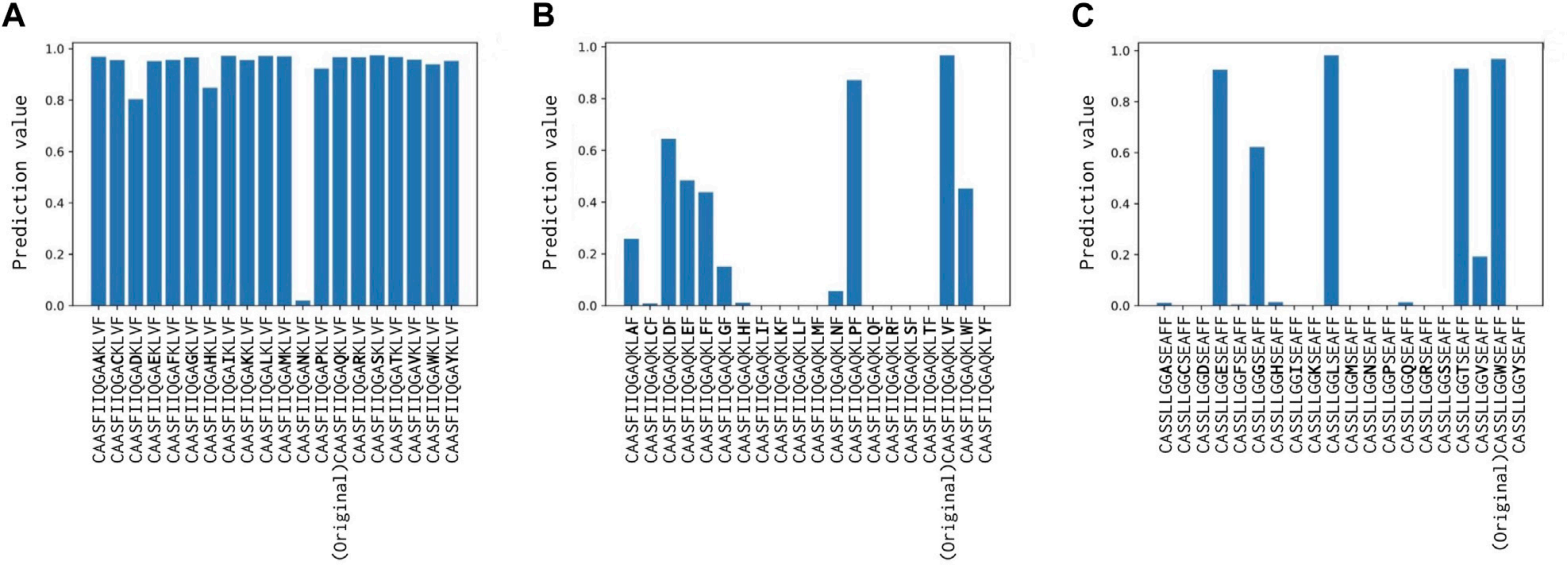
**(A)** Prediction change in Glutamine (Q) of CDR3*α* from CAASFIIQGAQKLVF to CAASFIIQGA (*)KLVF, where (*) denotes a substituted amino acid position. **(B)** Prediction change in Valine (V) of CDR3*α* from CAASFIIQGAQKLVF to CAASFIIQGAQKL (*)F. **(C)** Prediction change in Tryptophan (W) of CDR3*β* from CASSLLGGWSEAFF to CASSLLGG (*)SEAFF. Notably, only Valine (V) of CDR3*α* has two H-bonds with the internal CDR3 alpha chain, and hence, altering it has the most substantial impact.

When the 14th Val(V) of CDR3*α* was altered, the predictions experienced the most substantial impact, with the “unbound” prediction typically falling below 0.9 (Figure 6), probably because this attended residue has two H-bonds with CDR. Changes to the 11th Gln(Q) of the *α*-chain had a relatively minor effect on predictions, whereas alterations to the 9th Trp(W) of the *β*-chain modify predictions while maintaining positive predictions with various amino acid substitutions. These results can be also confirmed by Figure 5 and support our hypothesis that the internal H-bonded structure of CDR3 is crucial for peptide binding.

In the 1AO7 (before mutation) and 4FTV (after mutation) structures, Cole et al. (2013) identified that mutations in the four residues of the CDR3*β* chain of TCR enhanced binding to the peptide by nearly 1,000-fold. We evaluated how predicted y-values change when these amino acid residues are substituted, focusing on the two structures with mutations. As shown in Table 8, the CDR3*α* sequence of 1AO7 and 4FTV is CAVTTDSWG, with CDR3*β* for 1AO7 being CASRPGLAGGRP and for 4FTV being CASRPGLMSAQP. The 4FTV mutation was from AGGR to MSQP, with the 8th to 11th residues enhancing affinity. Remarkably, our model successfully focused on the mutated residues in 10th Ala(A) and 11th Gln(Q) of the *β*-chain, although the model predicted both of them as positive. Furthermore, Cole et al. (2013) posited that the mutation led to the loss of one hydrogen bond with the peptide, but the overall affinity was stronger after the mutation, suggesting an indirect contribution to the binding, except for the TCR–peptide H-bonds. This finding also should reinforce our assertion that the internal H-bonded structure of CDR3s is essential for peptide binding and reinforces the biological significance of attention values.

## Discussion

### Interpretation of the prediction in protein sequences

Prediction results of machine learning models are generally difficult to interpret, but when a model is used for binding predictions of biological sequences, the interpretability of the neural network model is essential. Highlighting residue positions is useful for understanding model predictions and is critical for utilizing later-stage applications. Our work, the cross-TCR-interpreter, attempts to enable this interpretation with the attention layer.

Unexpectedly, we found that CDR residues with large attention values in our ML model did not necessarily interact directly with peptide residues, as statistically shown in Table 6. Our result suggests that the ratio of hydrogen bond formation between CDR3s and a peptide can be relatively small yet result in positive predictions in the model.

Instead, the TCR residues with large attention values appeared to stabilize a specific loop conformation required for peptide binding by forming H bonds within CDR3s. Accordingly, researchers have observed that residues that comprise the H-bond network within TCR may be evolutionarily conserved (Garcia et al., 1996; Andrade et al., 2019), and the internal organization of the interface plays an important role in protein–protein interactions (Reichmann et al., 2005; Rauf et al., 2009). Our findings suggest that certain residues may be oriented in a specific direction with internal H-bonds, and the attention layer may emphasize their importance in TCRs in terms of binding stability.

Our findings also showed that the average distance between the TCR and peptide in 3D decreased when the attention value on the peptide side of attention was large. This may be because the peptide, being a short sequence, has a limited contribution to TCR binding that is related solely to distance. However, the larger attention values on the TCR side did not necessarily correspond to smaller distances in the 3D structure, potentially because TCRs are longer and more complicated in their binding role.

Not all the sequence paired data were available with a 3D structure, and we knew that the number was small, but we experimented with as many available structures as possible. We used the 3D structure as the confirmation of attention layer interpretation. In future investigations, it may be possible to use a different machine learning model such as a meaningful perturbation method on exhaustively collected sequences.

### Model limitations due to the dataset and difficulties associated with the recent data and COVID-19 dataset

In our investigation of the TCR–pMHC interaction, we simplified our focus to the CDR3 region of TCR and its peptides. This approach offers computational efficiency and enhanced interpretability by emphasizing the most variable and antigen-specific regions. Furthermore, given the data limitation to the experimental data on the whole sequences, it offers some advantages over the methods required to have the whole sequences. However, this narrowed scope might miss out on integral information from the complete TCR and MHC, potentially leading to overlooked critical interactions vital for binding. For a more comprehensive view of the entire binding mechanism, methods such as molecular simulations might be more suitable, although they are computationally demanding. Our model, similar to other models discussed in the Introduction section, captures a specific aspect of a complex biological process, and its utility must be contextualized based on research objectives and available resources.

Furthermore, our analysis and results might, admittedly, raise several questions regarding the interpretability of attention values observed between the TCR and peptide. The diverse sampling of TCR compared to the peptide samples in our dataset might influence the apparent association of significant attention with structural properties such as hydrogen bond formation primarily within TCR. This imbalance could help explain why our model predominantly associates the presence of certain TCR residues with reactivity against a specific peptide, rather than assigning weight to peptide-side attention.

**FIGURE 7:**
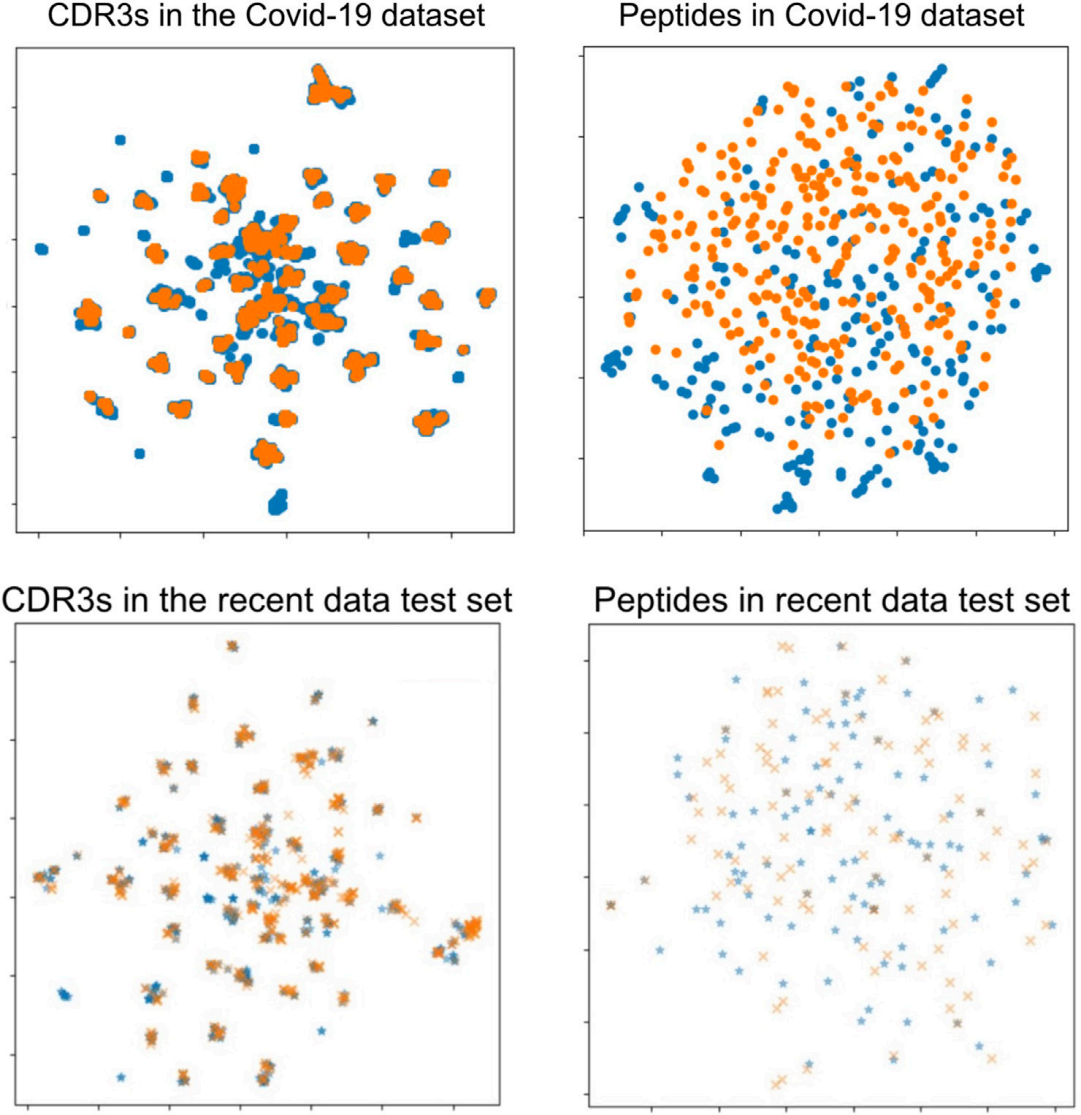
UMAP visualizations of the sequence distance maps of TCRs and peptides in the COVID-19 dataset (upper two figures) and the recent data test set (lower two figures). Each point shows a sequence datum, and the two colors on the peptide side (right two figures) show minimal overlap, indicating different peptides in those data datasets. Left: TCR sequence (CDR3*αβ*) visualization. Right: peptide sequence visualization. Orange points: the COVID-19 dataset or the recent data test set. Blue points: the combined data dataset.

The approach to generating negative data in benchmark datasets may skew our test dataset to resemble our training data more than would be typical in real-world prospective evaluation scenarios. This is indicated by the inferior performance of the recent data test set scores and the COVID-19 dataset results. The difficulty of prediction in the recent data test set and the COVID-19 data was not caused by differences in TCRs but, instead, by differences in peptides between the COVID-19 data and baseline data. We plotted the sequence–sequence pairwise distance matrix with UMAP dimension reduction, as shown in Figure 7. There was no difference in the distribution of TCRs between the data on combined data dataset and the COVID-19 data, whereas there was a substantial difference in the distribution of peptides. This discussion is also supported by previous studies on TCR predictions (Moris et al., 2021; Weber et al., 2021; Essaghir et al., 2022), in which the authors stated that generalization and extrapolation to unseen epitopes remain challenging.

In addition, when the COVID-19 dataset was modified to a positive ratio of 20%, the model ROC AUC value was 0.5881 and the average precision score was 0.2305. When we set the threshold giving the maximum F1-score, the precision was 0.2892 and the recall was 0.60. The positive ratio affected the performance of the model evaluation.

### Difficulties associated with unseen data

Additionally, we sought to evaluate the performance of our model specifically on data records involving unseen peptides or different TCRs within the benchmark test set of McPAS and VDJdb data. This was done either by removing the records of peptides of the training dataset or by removing similar TCRs from the test dataset.

Although the majority of the peptides were already present in the training data, we identified a subset of 46 records (14 positives) for McPAS and 16 records (eight positives) for VDJdb that involved unseen peptides (the numbers, 46 and 16, are also shown in Figure 2). The ROC AUC scores for these unseen peptide records were 0.721 for McPAS and 0.719 for VDJdb, which were a lot lower than the scores given in Tables 3, 4 for records involving observed peptides. This performance gain when evaluating the model on already observed peptides was also observed in the recent data test set experiment.

**FIGURE 8:**
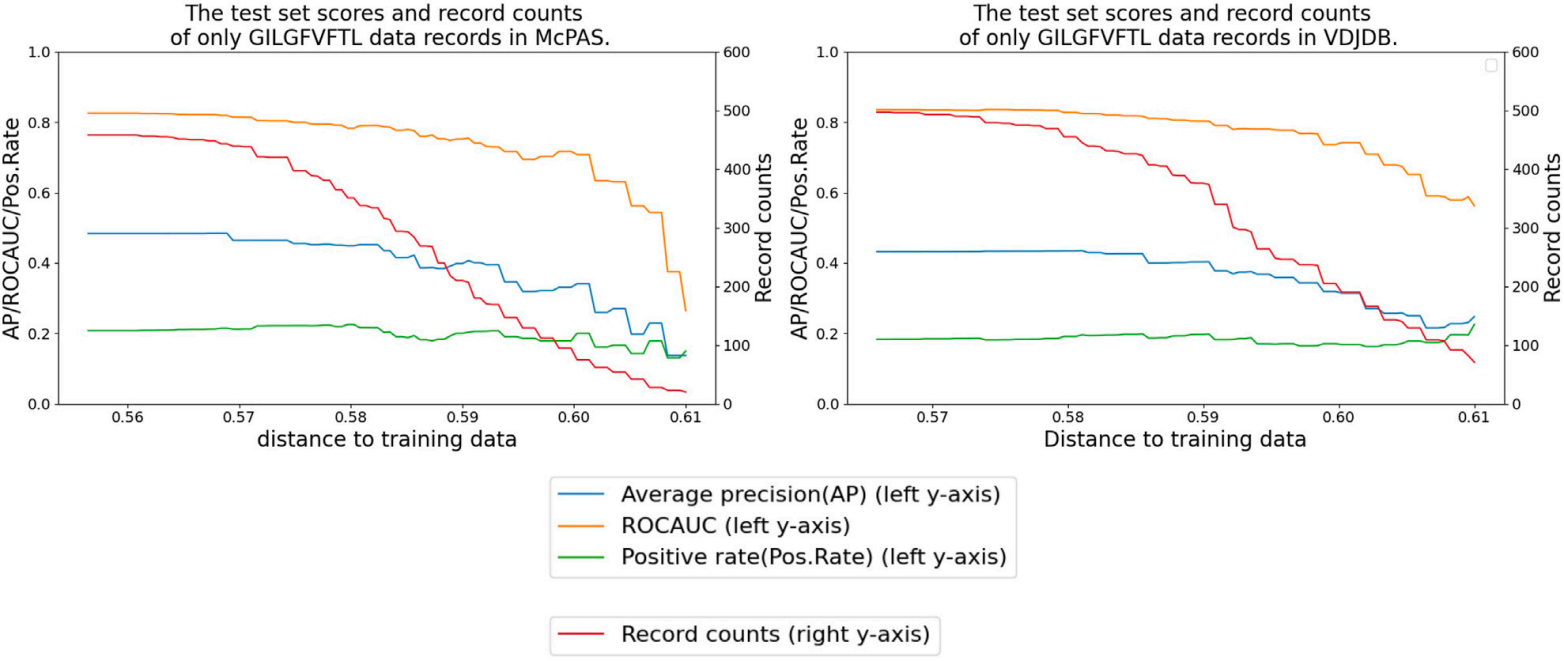
Performance delegation of the peptide GILGFVFTL by removing similar TCR records from the test set. The x-axis shows the distance threshold. For instance, if the x-value is 0.58, no test data less than that distance are used for evaluation.

Figure 8 shows that our performance metrics indicated a decrease when we refined the test dataset by eliminating any test records involving TCRs that exhibit a distance greater than a certain threshold value from the TCRs present in the training set. This trend underscores the sensitivity of the model to the diversity and distribution of TCRs in the test data.

## Conclusion

Our study presents a computational approach for predicting TCR binding to specific ligand peptides. Our study predicted the TCR–pMHC interaction with the cross-attention mechanism and analyzed the available protein structures comprehensively to gain new insights into TCR–peptide functional relationships.

By incorporating an attention layer based on language models, our machine learning model achieved competitive performance on a benchmark dataset of the TCR–pMHC interaction, although it confronted enduring challenges with the COVID-19 dataset and the recent data test set.

Our analysis of the model allowed us to associate neural network weights with protein 3D structure datasets, identify statistically significant properties of largely attended residues, and detail the binding principle through the visualization and analysis of the cross-attention layer, the source–target attention layer.

The statistical analysis of the attention layer on the structural data revealed that the largely attended residues were more likely to contact their own CDR3 than normal residues, thereby providing new insights into the CDR3–peptide binding mechanisms. Proteins create hydrogen bonds to form special structures and may play special roles when a peptide is conditioned to react with them.

## Data Availability

Publicly available datasets were analyzed in this study. These data can be found at: https://github.com/kyoheikoyama/TCRPrediction/tree/main/data.

## References

[B1] Akiba T. Sano S. Yanase T. Ohta T. Koyama M. (2019). “Optuna: a next-generation hyperparameter optimization framework,” in Proceedings of the 25th ACM SIGKDD international conference on knowledge discovery and data mining, 2623–2631.

[B2] Andrade M. Pontes C. Treptow W. (2019). Coevolutive, evolutive and stochastic information in protein-protein interactions. Comput. Struct. Biotechnol. J. 17, 1429–1435. 10.1016/j.csbj.2019.10.005 31871588 PMC6906720

[B3] Berman H. Henrick K. Nakamura H. (2003). Announcing the worldwide protein data bank. Nat. Struct. Mol. Biol. 10, 980. 10.1038/nsb1203-980 14634627

[B4] Chapman B. Chang J. (2000). Biopython: Python tools for computational biology. ACM Sigbio Newsl. 20, 15–19. 10.1145/360262.360268

[B5] Chen J. Yang L. Raman K. Bendersky M. Yeh J.-J. Zhou Y. (2020). Dipair: fast and accurate distillation for trillion-scale text matching and pair modeling. *arXiv preprint arXiv:2010.03099* .

[B6] Cole D. K. Sami M. Scott D. R. Rizkallah P. J. Borbulevych O. Y. Todorov P. T. (2013). Increased peptide contacts govern high affinity binding of a modified tcr whilst maintaining a native pmhc docking mode. Front. Immunol. 4, 168. 10.3389/fimmu.2013.00168 23805144 PMC3693486

[B7] Dash P. Fiore-Gartland A. J. Hertz T. Wang G. C. Sharma S. Souquette A. (2017). Quantifiable predictive features define epitope-specific T cell receptor repertoires. Nature 547, 89–93. 10.1038/nature22383 28636592 PMC5616171

[B8] Devlin J. Chang M.-W. Lee K. Toutanova K. (2018). Bert: pre-training of deep bidirectional transformers for language understanding. *arXiv preprint arXiv:1810.04805* .

[B9] Dunbar J. Deane C. M. (2016). Anarci: antigen receptor numbering and receptor classification. Bioinformatics 32, 298–300. 10.1093/bioinformatics/btv552 26424857 PMC4708101

[B10] Essaghir A. Sathiyamoorthy N. K. Smyth P. Postelnicu A. Ghiviriga S. Ghita A. (2022). T-cell receptor specific protein language model for prediction and interpretation of epitope binding (protlm. tcr). New York, NY: Cold Spring Harbor Laboratory (CSHL). bioRxiv. 10.1101/2022.11.28.518167

[B11] Gao Y. Gao Y. Fan Y. Zhu C. Wei Z. Zhou C. (2023). Pan-peptide meta learning for t-cell receptor–antigen binding recognition. Nat. Mach. Intell. 5, 236–249. 10.1038/s42256-023-00619-3

[B12] Garcia K. C. Degano M. Stanfield R. L. Brunmark A. Jackson M. R. Peterson P. A. (1996). An *αβ* t cell receptor structure at 2.5 å and its orientation in the tcr-mhc complex. Science 274, 209–219. 10.1126/science.274.5285.209 8824178

[B14] Gheini M. Ren X. May J. (2021). “Cross-attention is all you need: adapting pretrained Transformers for machine translation,” in Proceedings of the 2021 conference on empirical methods in natural language processing, 1754–1765. 10.18653/v1/2021.emnlp-main.132

[B15] Gowthaman R. Pierce B. G. (2019). Tcr3d: the t cell receptor structural repertoire database. Bioinformatics 35, 5323–5325. 10.1093/bioinformatics/btz517 31240309 PMC6954642

[B16] Hao Y. Dong L. Wei F. Xu K. (2021). Self-attention attribution: interpreting information interactions inside transformer. Proc. AAAI Conf. Artif. Intell. 35, 12963–12971. 10.1609/aaai.v35i14.17533

[B17] Honda S. Koyama K. Kotaro K. (2020). “Cross attentive antibody-antigen interaction prediction with multi-task learning,” in ICML 2020 workshop on computational biology (WCB).

[B18] Koyama K. Kamiya K. Shimada K. (2020). Cross attention dti: drug-target interaction prediction with cross attention module in the blind evaluation setup. BIOKDD2020.

[B19] Lee K.-H. Chen X. Hua G. Hu H. He X. (2018). “Stacked cross attention for image-text matching,” in Proceedings of the European conference on computer vision (Munich, Germany: ECCV), 201–216.

[B20] Lu T. Zhang Z. Zhu J. Wang Y. Jiang P. Xiao X. (2021a). Deep learning-based prediction of the t cell receptor–antigen binding specificity. Nat. Mach. Intell. 3, 864–875. 10.1038/s42256-021-00383-2 36003885 PMC9396750

[B21] Lu X. Hosono Y. Nagae M. Ishizuka S. Ishikawa E. Motooka D. (2021b). Identification of conserved SARS-CoV-2 spike epitopes that expand public cTfh clonotypes in mild COVID-19 patients. J. Exp. Med. 218, e20211327. 10.1084/jem.20211327 34647971 PMC8641254

[B22] Mahajan S. Yan Z. Jespersen M. C. Jensen K. K. Marcatili P. Nielsen M. (2019). Benchmark datasets of immune receptor-epitope structural complexes. BMC Bioinforma. 20, 490–497. 10.1186/s12859-019-3109-6 PMC6785892 31601176

[B23] Montemurro A. Schuster V. Povlsen H. R. Bentzen A. K. Jurtz V. Chronister W. D. (2021). NetTCR-2.0 enables accurate prediction of TCR-peptide binding by using paired TCR*α* and *β* sequence data. Commun. Biol. 4, 1–13. 10.1038/s42003-021-02610-3 34508155 PMC8433451

[B24] Moris P. De Pauw J. Postovskaya A. Gielis S. De Neuter N. Bittremieux W. (2021). Current challenges for unseen-epitope tcr interaction prediction and a new perspective derived from image classification. Briefings Bioinforma. 22, bbaa318. 10.1093/bib/bbaa318 PMC8294552 33346826

[B25] Parthasarathy S. Sundaram S. (2021). “Detecting expressions with multimodal transformers,” in 2021 IEEE Spoken Language Technology Workshop (SLT) (IEEE), 636–643.

[B26] Rauf S. M. A. Ismael M. Sahu K. K. Suzuki A. Sahnoun R. Koyama M. (2009). A graph theoretical approach to the effect of mutation on the flexibility of the dna binding domain of p53 protein. Chem. Pap. 63, 654–661. 10.2478/s11696-009-0068-9

[B27] Reichmann D. Rahat O. Albeck S. Meged R. Dym O. Schreiber G. (2005). The modular architecture of protein-protein binding interfaces. Proc. Natl. Acad. Sci. 102, 57–62. 10.1073/pnas.0407280102 15618400 PMC544062

[B28] Rogers A. Kovaleva O. Rumshisky A. (2020). A primer in bertology: what we know about how bert works. Trans. Assoc. Comput. Linguistics 8, 842–866. 10.1162/tacl_a_00349

[B29] Schrödinger L. L. C. DeLano W. (2020). Pymol. Available at: http://www.pymol.org/pymol.

[B30] Shugay M. Bagaev D. V. Zvyagin I. V. Vroomans R. M. Crawford J. C. Dolton G. (2018). Vdjdb: a curated database of t-cell receptor sequences with known antigen specificity. Nucleic acids Res. 46, D419–D427. 10.1093/nar/gkx760 28977646 PMC5753233

[B31] Sidhom J.-W. Larman H. B. Pardoll D. M. Baras A. S. (2021). DeepTCR is a deep learning framework for revealing sequence concepts within T-cell repertoires. Nat. Commun. 12, 1605–1612. 10.1038/s41467-021-21879-w 33707415 PMC7952906

[B32] Sievers F. Wilm A. Dineen D. Gibson T. J. Karplus K. Li W. (2011). Fast, scalable generation of high-quality protein multiple sequence alignments using clustal omega. Mol. Syst. Biol. 7, 539. 10.1038/msb.2011.75 21988835 PMC3261699

[B33] Springer I. Besser H. Tickotsky-Moskovitz N. Dvorkin S. Louzoun Y. (2020). Prediction of specific TCR-peptide binding from large dictionaries of TCR-peptide pairs. Front. Immunol. 11, 1803. 10.3389/fimmu.2020.01803 32983088 PMC7477042

[B34] Springer I. Tickotsky N. Louzoun Y. (2021). Contribution of T cell receptor alpha and beta CDR3, MHC typing, V and J genes to peptide binding prediction. Front. Immunol. 12, 664514. 10.3389/fimmu.2021.664514 33981311 PMC8107833

[B35] Tickotsky N. Sagiv T. Prilusky J. Shifrut E. Friedman N. (2017). Mcpas-tcr: a manually curated catalogue of pathology-associated t cell receptor sequences. Bioinformatics 33, 2924–2929. 10.1093/bioinformatics/btx286 28481982

[B36] Vaswani A. Shazeer N. Parmar N. Uszkoreit J. Jones L. Kaiser Ł. (2017). Attention is all you need. Adv. neural Inf. Process. Syst. 30 . 10.48550/arXiv.1706.03762

[B37] Voita E. Talbot D. Moiseev F. Sennrich R. Titov I. (2019). “Analyzing multi-head self-attention: specialized heads do the heavy lifting, the rest can be pruned,” in Proceedings of the 57th annual meeting of the association for computational linguistics (Florence, Italy: Association for Computational Linguistics), 5797–5808. 10.18653/v1/P19-1580

[B38] Wallace A. C. Laskowski R. A. Thornton J. M. (1995). Ligplot: a program to generate schematic diagrams of protein-ligand interactions. Protein Eng. Des. Sel. 8, 127–134. 10.1093/protein/8.2.127 7630882

[B39] Weber A. Born J. Rodriguez Martínez M. (2021). Titan: T-cell receptor specificity prediction with bimodal attention networks. Bioinformatics 37, i237–i244. 10.1093/bioinformatics/btab294 34252922 PMC8275323

[B40] Wu K. Yost K. E. Daniel B. Belk J. A. Xia Y. Egawa T. (2021). TCR-BERT: learning the grammar of t-cell receptors for flexible antigen-xbinding analyses. bioRxiv, 11.18.469186.

[B13] 10x Genomics (2019). A new way of exploring immunity–linking highly multiplexed antigen recognition to immune repertoire and phenotype. Tech. Rep.

[B41] Xu Y. Qian X. Tong Y. Li F. Wang K. Zhang X. (2022). AttnTAP: a dual-input framework incorporating the attention mechanism for accurately predicting TCR-peptide binding. Front. Genet. 13, 942491. 10.3389/fgene.2022.942491 36072653 PMC9441555

[B42] Xu Z. Luo M. Lin W. Xue G. Wang P. Jin X. (2021). Dlptcr: an ensemble deep learning framework for predicting immunogenic peptide recognized by t cell receptor. Briefings Bioinforma. 22, bbab335. 10.1093/bib/bbab335 34415016

[B43] Yang X. Chen G. Weng N.-p. Mariuzza R. A. (2017). Structural basis for clonal diversity of the human T-cell response to a dominant influenza virus epitope. J. Biol. Chem. 292, 18618–18627. 10.1074/jbc.M117.810382 28931605 PMC5683187

