# Supplementary material for: Attention network for predicting T-cell receptor–peptide binding can associate attention with interpretable protein structural properties
Source: Front Bioinform. 2023 Dec 18;3:1274599. doi: 10.3389/fbinf.2023.1274599 (PMC10759225; doi:10.3389/fbinf.2023.1274599)
Supplement: Supplementary file 1 [file DataSheet1.PDF]

Supplementary Material

HYPERPARAMETERS OF THE MODEL

The parameter of the model was optimized by using a hyperparameter optimization package, Optuna. The best parameters are $batch\_size$ = 2949, d_ff of the final MLP layer = 84, dim in the model = 256, $dropout\_rate$ = 7.651e-05, and $learning\_rate$ = 9.387e-05.

The number of transformer layers for self-attention is two on each side of the TCR and the peptide. The n_head of the transformer encoder is four. The number of cross-attention layers is one. The maximum length of TCRα and TCRβ for the padding was 62, and that of the peptide was 26. Although an intensive and exhaustive search may have different results, initial searches on pre-training and transfer learning did not contribute to improving the final score.

COMPARISON OF OCCURRENCE OF AMINO ACID TYPES OF RESIDUES IN THE LARGE AND SMALL ATTENTION GROUPS

In general, TCRs have, in order of abundance, serine(S), alanine (A), and phenylalanine (F). On the other hand, TCRs with large attention values have valine (V), glycine (G), and glutamic acid (E). As the right panel of Figure S1 shows, methionine (M), valine (V), and lysine (K) tend to have relatively large attention values. The side chains of M and V are known as polarity-free and belong to the aliphatic group.

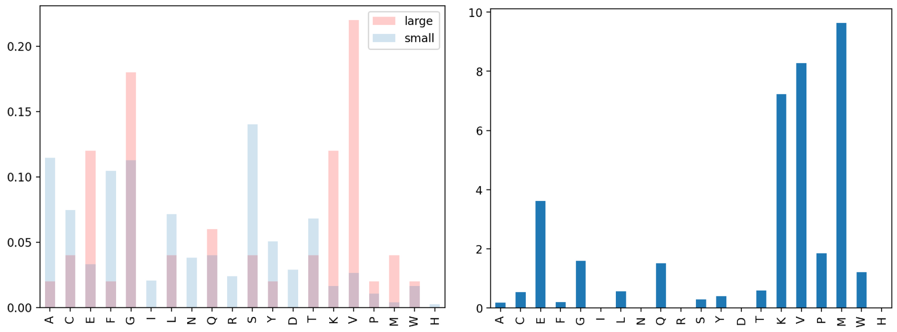

Figure S1: Left: Residue type distribution of large attention TCR group and small attention TCR group. Right: the value of the large group divided by the value of the small group, indicating the occurrence tendency of large attention value.

INFULUENCE OF THE CHANGE OF THE γ FACTOR

The attention values were considered ”large” when they exceeded the threshold of MEAN + 5.5 STD on the peptide side and MEAN + 4.5 STD on the TCR side (4.5 and 5.5 are γ factors). Approximately 20% of the residues were identified as large on each side, using γ as a result of the total sum of the four heads Table S1.

Table S1. Count of large or small attention residue groups when changing the factor γ. Four heads are merged.

	**TCRs Large Attention**	**TCRs Small Attention**	**Peptides Large Attention**	**Peptides Small Attention**
**count (γ=2)**	569	468	372	65
**count (γ=2.5)**	441	596	325	112
**count (γ=3))**	364	673	264	173
**count (γ=3.5**	299	738	219	218
**count (γ=4))**	244	793	172	265
**count (γ=4.5**	196	841	137	300
**count (γ=5))**	153	884	111	326
**count (γ=5.5**	118	919	81	356
**count (γ=6))**	97	940	48	389

ADDITIONAL DATASET STATISTICS

Unique count statistics are shown in Table S2. The interaction column means the unique count of pairs of $\{$CDR3α, CDR3β, Peptide$\}$. The duplication count means the number of unique data that is shared between training and test sets.

Additionally, Figure S2 shows the distribution of the length for each dataset.

Table S2. Unique count statistics.

Dataset name	CDR3α	CDR3β	Interaction
McPAS, training	2423	2560	23363
McPAS, test	718	714	4729
McPAS, duplication count	218	198	0
VDJdb-without10x, training	2151	2171	19526
VDJdb-without10x, test	570	572	4010
VDJdb, duplication count	198	196	0
The combined data dataset	17954	19162	119046
The recent data test set	4782	5174	33360
The duplication count of the two above	444	148	0

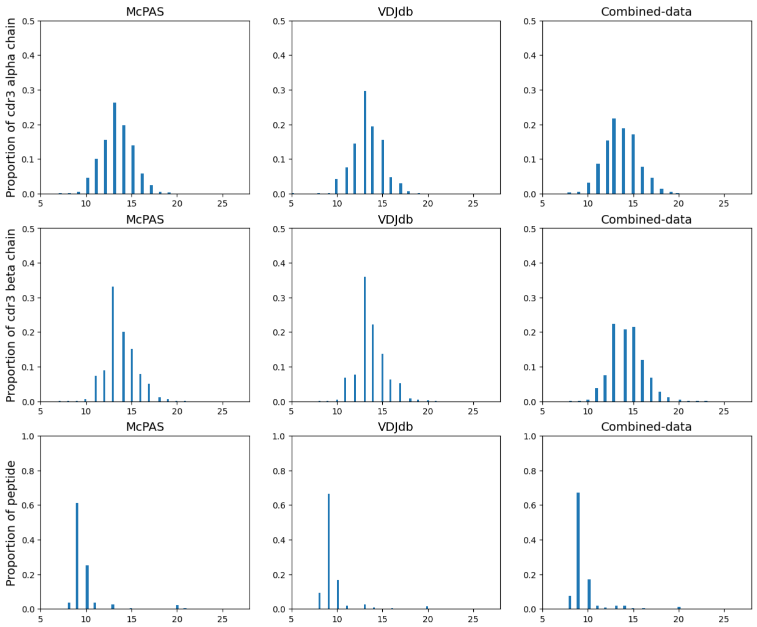

Figure S2: The distribution of the length for each dataset.

ATTENTION CAN CAPTURE THE SIGN OF POSITIVE BINDING

In a minor experiment, we tested the attention mechanism of our model and its effectiveness using mock data. We added two Alanine (A) residues to the end of TCR alpha for every positive pair, indicating a clear and obvious sign of positive binding. After training the model on this mock data from McPAS, it achieved the ROCAUC score of 1.0. Upon visualizing the attention layer of the trained model, we found that the sign of positive interactions was indeed given large attention values, as shown in Figure S3. Hence, we used attention values as a tool to interpret the binding mechanism.

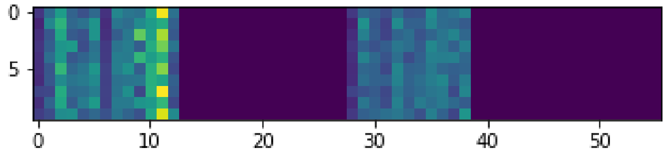

Figure S3: Attention values visualization on one of the mock dataset pairs. The position of the 11th residue was the first Alanine added to the TCR alpha chain, and it had large attention values. X-axis: Residues of TCRs. Y-axis: Residues of a peptide. Dark blue indicates zero attention values (padding) and yellow indicates large values.

COMPARISON OF MODELS ON THE RECENT TEST DATASET

To address the query about the performance of our model trained on the combined data dataset in comparison to models trained on smaller datasets, we compared three models: The ”combined-data-trained model”, the ”McPAS-model-trained” and the ”VDJdb-model”. The question was essentially about whether or not training on a larger combined dataset degrades the model’s performance.

Each model was evaluated on a recent test dataset. The combined-data-trained model was developed using a larger training dataset that combines data from both the McPAS and VDJdb databases. In contrast, the McPAS-model and VDJdb-model were trained using smaller, individual datasets.

The results are as follows:

- **Combined-data-trained model:** This model achieved the average precision (AP) of 0.1855 and an ROCAUC score of 0.5362 as stated in the Table 5 of the manuscript.
- **McPAS-trained model:** Evaluated scores were 0.1797 for AP and 0.5292 for ROCAUC. These scores were slightly lower than the combined-data-trained model.
- **VDJdb-trained model:** This model returned an ROCAUC of 0.5235, which is below the combined model. However, its AP was 0.1860, comparable to the combined-data-trained model. Given that the recent test dataset originates from the VDJdb database, this model might have had a slight advantage.

The combined-data-trained model performed at least as well as the VDJdb-trained model and outperformed the McPAS-model. It's crucial to note that the "recent data test set" for the combined dataset is temporally distinct from its training dataset, providing a stringent testing condition. In contrast, test sets for VDJdb and McPAS were not temporally distinct. We believe this temporal distinctness explains the lower scores on the recent data test set, rather than the influence of the larger training dataset.

In conclusion, training on a larger combined dataset does not degrade the model's performance. In fact, the combined-data-trained model proves to be competitive, if not superior, when compared to models trained on individual datasets.

PDB IDS FOR TCR-PEPTIDE CRYSTAL

There are 39 PDB IDs for the analysis out of the 80 TCR-peptide crystal structures of PDB. 9 PDB IDs were not included in the combined data dataset, whereas 30 PDB IDs were included.

- 55 PDBIDs before processing:

- 2VLK, 2ICW, 5WKF, 2VLJ, 3PQY, 4MJI, 4P2Q, 2YPL, 5BRZ, 6RPB, 1J8H, 4P2R, 5MEN, 3MV8, 4OZF, 3VXR, 3VXS, 4OZG, 3MV9, 5TEZ, 2J8U, 6Q3S, 4JRX, 5WLG, 3VXU, 1U3H, 4JRY, 4Z7V, 2UWE, 1LP9, 3W0W, 6AVF, 2BNQ, 4JFE, 4JFD, 3QIU, 6AVG, 2Z31, 2BNR, 5ISZ, 5KS9, 3MV7, 3MBE, 4OZH, 2NX5, 5NHT, 4QOK, 5D2L, 1D9K, 1FYT, 4P2O, 5WKH, 6EQB, 2VLR, 6EQA

- 47 PDBIDs having no identical sequences:

- 2VLK, 2ICW, 5WKF, 3PQY, 4MJI, 4P2Q, 2YPL, 5BRZ, 6RPB, 1J8H, 4P2R, 5MEN, 3MV8, 4OZF, 3VXR, 3VXS, 4OZG, 5TEZ, 2J8U, 6Q3S, 4JRX, 5WLG, 3VXU, 1U3H, 4JRY, 4Z7V, 6AVF, 4JFE, 4JFD, 3QIU, 6AVG, 2Z31, 2BNR, 5ISZ, 5KS9, 3MBE, 4OZH, 2NX5, 5NHT, 4QOK, 5D2L, 1D9K, 4P2O, 5WKH, 6EQB, 2VLR, 6EQA

- 39 PDB IDs having positive predictions:

- 2VLK, 5WKF, 3PQY, 4MJI, 4P2Q, 2YPL, 1J8H, 4P2R, 5MEN, 3MV8, 4OZF, 3VXR, 3VXS, 4OZG, 5TEZ, 2J8U, 6Q3S, 4JRX, 3VXU, 1U3H, 4JRY, 4Z7V, 4JFE, 4JFD, 3QIU, 2Z31, 2BNR, 3MBE, 4OZH, 2NX5, 5NHT, 4QOK, 5D2L, 1D9K, 4P2O, 5WKH, 6EQB, 2VLR, 6EQA

- 9 PDB IDs that are not in the combined data dataset:

- 5MEN, 2J8U, 3VXU, 4JFE, 4JFD, 5NHT, 4QOK, 6EQB, 6EQA

S PROTEIN SEQUENCE OF COVID-19

- mfvflvllpl vssqcvnltt rtqlppaytn sftrgvyypd kvfrssvlhs tqdlflpffs nvtwfhaihv sgtngtkrfd npvlpfndgv yfasteksni irgwifgttl dsktqslliv nnatnvvikv cefqfcndpf lgvyyhknnk swmesefrvy ssannctfey vsqpflmdle gkqgnfknlr efvfknidgy fkiyskhtpi nlvrdlpqgf saleplvdlp iginitrfqt llalhrsylt pgdsssgwta gaaayyvgyl qprtfllkyn engtitdavd caldplsetk ctlksftvek giyqtsnfrv qptesivrfp nitnlcpfge vfnatrfasv yawnrkrisn cvadysvlyn sasfstfkcy gvsptklndl cftnvyadsf virgdevrqi apgqtgkiad ynyklpddft gcviawnsnn ldskvggnyn ylyrlfrksn lkpferdist eiyqagstpc ngvegfncyf plqsygfqpt ngvgyqpyrv vvlsfellha patvcgpkks tnlvknkcvn fnfngltgtg vltesnkkfl pfqqfgrdia dttdavrdpq tleilditpc sfggvsvitp gtntsnqvav lyqdvnctev pvaihadqlt ptwrvystgs nvfqtragcl igaehvnnsy ecdipigagi casyqtqtns prrarsvasq siiaytmslg aensvaysnn siaiptnfti svtteilpvs mtktsvdctm yicgdstecs nlllqygsfc tqlnraltgi aveqdkntqe vfaqvkqiyk tppikdfggf nfsqilpdps kpskrsfied llfnkvtlad agfikqygdc lgdiaardli caqkfngltv lppllldemi aqytsallag titsgwtfga gaalqipfam qmayrfngig vtqnvlyenq klianqfnsa igkiqdslss tasalgklqd vvnqnaqaln tlvkqlssnf gaissvlndi lsrldkveae vqidrlitgr lqslqtyvtq qliraaeira sanlaatkms ecvlgqskrv dfcgkgyhlm sfpqsaphgv vflhvtyvpa qeknfttapa ichdgkahfp regvfvsngt hwfvtqrnfy epqiittdnt fvsgncdvvi givnntvydp lqpeldsfke eldkyfknht spdvdlgdis ginasvvniq keidrlneva knlneslidl qelgkyeqyi kwpwyiwlgf iagliaivmv timlccmtsc csclkgccsc gscckfdedd sepvlkgvkl

STATISTICAL TEST RESULTS OF STRUCTURES USING DIFFERENT ATTENTION HEADS

The results of the paired t-tests are shown. Although each head was analyzed equally and separately, they showed different results (Table S3, Table S4).

Table S3. Peptide side attention analysis on all heads. In a cell, the left number is the mean value over the PDBIDs and the right number is the STD value.

Property	Large Attention [1]	Small Attention [1]	p-value	Head
Closest distance to peptide (Å)	4.1233±1.3970	5.0796±1.1194	0.0703	0
Number of H-bonds formed	1.6667±1.5986	2.0125±0.7579	0.84	0
H-bonded to any CDR3 residue	0.0833±0.2764	0.1801±0.1086	0.409	0
H-bonded to any peptide residue	0.0833±0.2764	0.0656±0.1218	1	0
H-bonded to any TCR residue	0.3333±0.4714	0.2559±0.1198	0.552	0
H-bonded to any non-CDR3 TCR residue	0.2500±0.4330	0.1167±0.1116	0.318	0
In the edge [2]	0.2500±0.4330	0.5637±0.0951	0.0504	0
Closest distance to peptide (Å)	5.1794±2.4525	5.0293±1.0674	0.895	1
Number of H-bonds formed	2.4348±1.7087	1.9672±0.7934	0.154	1
H-bonded to any CDR3 residue	0.3261±0.4570	0.1710±0.0996	0.215	1
H-bonded to any peptide residue	0.0000±0.0000	0.0673±0.1226	0.0711	1
H-bonded to any TCR residue	0.4130±0.4812	0.2499±0.1138	0.168	1
H-bonded to any non-CDR3 TCR residue	0.1739±0.3790	0.1162±0.1197	0.457	1
In the edge [2]	0.4783±0.4995	0.5610±0.1018	0.566	1
Closest distance to peptide (Å)	4.5241±1.8359	5.0844±1.0686	0.0771	2
Number of H-bonds formed	1.6304±1.0448	2.0183±0.7681	0.478	2
H-bonded to any CDR3 residue	0.1522±0.3437	0.1791±0.1188	0.653	2
H-bonded to any peptide residue	0.0435±0.2039	0.0659±0.1220	0.527	2
H-bonded to any TCR residue	0.2174±0.4125	0.2574±0.1175	0.827	2
H-bonded to any non-CDR3 TCR residue	0.0652±0.2238	0.1206±0.1151	0.664	2
In the edge [2]	0.4783±0.4773	0.5588±0.0997	0.688	2
Closest distance to peptide (Å)	3.7267±1.0947	5.1352±1.0994	5.15e-05	3
Number of H-bonds formed	1.7200±1.2496	2.0264±0.7809	0.503	3
H-bonded to any CDR3 residue	0.2800±0.4490	0.1714±0.0963	0.196	3
H-bonded to any peptide residue	0.1200±0.3250	0.0620±0.1108	0.31	3
H-bonded to any TCR residue	0.4000±0.4899	0.2491±0.1120	0.0866	3
H-bonded to any non-CDR3 TCR residue	0.1200±0.3250	0.1197±0.1162	0.697	3
In the edge [2]	0.2600±0.4271	0.5771±0.1191	0.00591	3
H-bonded to any peptide residue	0.0495±0.1443	0.0659±0.1206	0.458	all
H-bonded to any CDR3 residue	0.2050±0.3024	0.1682±0.0982	0.48	all
H-bonded to any TCR residue	0.3401±0.3714	0.2372±0.1184	0.151	all
H-bonded to any non-CDR3 TCR residue	0.1712±0.3112	0.1118±0.1283	0.355	all
In the edge [2]	0.4459±0.4097	0.5874±0.1232	0.0795	all
Closest distance to peptide (Å)	4.6398±1.7149	5.1926±1.2647	0.141	all
Number of H-bonds formed	2.1126±1.4959	2.0031±0.9051	0.668	all

1. Mean and standard deviation (for the 39 structures) of the proportion of residues that satisfy the property shown in the first column.
2. Three residues from the beginning and four from the end of the peptide. 3. In the last two properties, per-residue averages were used instead.

Table S4. TCR side attention analysis on all heads. In a cell, the left number is the mean and the right number is the STD over the PDBIDs.

Property	Large Attention [1]	Small Attention [1]	p-value	Head
H-bonded to any peptide residue	0.0256 ± 0.1581	0.0853 ± 0.0577	0.043	0
H-bonded to any CDR3 residue	0.6068 ± 0.4262	0.4135 ± 0.1061	0.0111	0
H-bonded to any TCR residue of own chain	0.7051 ± 0.4038	0.6408 ± 0.0888	0.353	0
H-bonded to any CDR3 residue of own chain	0.6068 ± 0.4262	0.3792 ± 0.0991	0.0027	0
H-bonded to any TCR residue of opposite chain	0.1111 ± 0.2833	0.1565 ± 0.0756	0.371	0
H-bonded to any CDR3 residue of opposite chain	0.0000 ± 0.0000	0.0642 ± 0.0673	8.37e-07	0
H-bonded to any TCR residue	0.7308 ± 0.3897	0.7193 ± 0.0903	0.864	0
H-bonded to any non-CDR3 TCR residue	0.1966 ± 0.3734	0.4479 ± 0.0738	0.000482	0
In the edge [2]	0.8376 ± 0.3235	0.5904 ± 0.0387	5.1e-05	0
Closest distance to peptide (Å)	9.1216 ± 3.1274	8.3528 ± 1.0097	0.119	0
Number of H-bonds formed	1.7479 ± 1.1003	2.0919 ± 0.6806	0.0397	0
H-bonded to any peptide residue	0.1453 ± 0.3162	0.0773 ± 0.0564	0.2	1
H-bonded to any CDR3 residue	0.5171 ± 0.4333	0.4147 ± 0.1077	0.183	1
H-bonded to any TCR residue of own chain	0.5427 ± 0.4315	0.6488 ± 0.0892	0.152	1
H-bonded to any CDR3 residue of own chain	0.5085 ± 0.4335	0.3817 ± 0.0999	0.0913	1
H-bonded to any TCR residue of opposite chain	0.1496 ± 0.3198	0.1510 ± 0.0714	0.979	1
H-bonded to any CDR3 residue of opposite chain	0.0171 ± 0.1054	0.0616 ± 0.0678	0.0467	1
H-bonded to any TCR residue	0.6368 ± 0.4480	0.7211 ± 0.0881	0.264	1
H-bonded to any non-CDR3 TCR residue	0.1581 ± 0.2995	0.4511 ± 0.0648	5.28e-07	1
In the edge [2]	0.5513 ± 0.4388	0.6055 ± 0.0443	0.477	1
Closest distance to peptide (Å)	7.7849 ± 3.3952	8.4412 ± 0.9215	0.15	1
Number of H-bonds formed	1.9957 ± 1.5568	2.0725 ± 0.6687	0.742	1
H-bonded to any peptide residue	0.1127 ± 0.2935	0.0805 ± 0.0585	0.654	2
H-bonded to any CDR3 residue	0.3775 ± 0.4470	0.4247 ± 0.0992	0.614	2
H-bonded to any TCR residue of own chain	0.4853 ± 0.4615	0.6499 ± 0.0851	0.067	2
H-bonded to any CDR3 residue of own chain	0.3480 ± 0.4379	0.3919 ± 0.0947	0.633	2
H-bonded to any TCR residue of opposite chain	0.1324 ± 0.3278	0.1557 ± 0.0707	0.759	2
H-bonded to any CDR3 residue of opposite chain	0.0588 ± 0.2353	0.0614 ± 0.0678	0.933	2
H-bonded to any TCR residue	0.5882 ± 0.4451	0.7249 ± 0.0874	0.127	2
H-bonded to any non-CDR3 TCR residue	0.3235 ± 0.4021	0.4399 ± 0.0748	0.144	2
In the edge [2]	0.4706 ± 0.4705	0.6060 ± 0.0390	0.126	2
Closest distance to peptide (Å)	7.9293 ± 4.0178	8.3982 ± 1.0153	0.588	2
Number of H-bonds formed	2.1765 ± 1.8190	2.0746 ± 0.6642	0.607	2
H-bonded to any peptide residue	0.0877 ± 0.2470	0.0818 ± 0.0572	0.909	3
H-bonded to any CDR3 residue	0.4342 ± 0.4318	0.4234 ± 0.1013	0.852	3
H-bonded to any TCR residue of own chain	0.5921 ± 0.4270	0.6499 ± 0.0865	0.466	3
H-bonded to any CDR3 residue of own chain	0.3947 ± 0.4161	0.3908 ± 0.0969	0.927	3
H-bonded to any TCR residue of opposite chain	0.2237 ± 0.2993	0.1476 ± 0.0693	0.116	3
H-bonded to any CDR3 residue of opposite chain	0.0526 ± 0.1916	0.0615 ± 0.0631	0.767	3
H-bonded to any TCR residue	0.6842 ± 0.3723	0.7236 ± 0.0884	0.57	3
H-bonded to any non-CDR3 TCR residue	0.3684 ± 0.3590	0.4391 ± 0.0678	0.263	3
In the edge [2]	0.7281 ± 0.3735	0.5962 ± 0.0430	0.0464	3
Closest distance to peptide (Å) [3]	8.6919 ± 3.5172	8.4100 ± 1.0378	0.597	3
Number of H-bonds formed [3]	2.1535 ± 1.4009	2.0690 ± 0.6701	0.622	3
H-bonded to any peptide residue	0.0862±0.1368	0.0805±0.0675	0.828	all
H-bonded to any CDR3 residue	0.4846±0.2216	0.4103±0.1040	0.0478	all
H-bonded to any TCR residue of own chain	0.6013±0.1999	0.6561±0.0880	0.117	all
H-bonded to any CDR3 residue of own chain	0.4643±0.2180	0.3752±0.0922	0.0107	all
H-bonded to any TCR residue of opposite chain	0.1679±0.1714	0.1497±0.0793	0.562	all
H-bonded to any CDR3 residue of opposite chain	0.0306±0.0857	0.0672±0.0743	0.0369	all
H-bonded to any TCR residue	0.6845±0.1650	0.7294±0.0880	0.0987	all
H-bonded to any non-CDR3 TCR residue	0.2940±0.1923	0.4672±0.0846	3.88e-05	all
In the edge [2]	0.6434±0.2064	0.5928±0.0570	0.218	all
Closest distance to peptide (Å) [3]	8.4072±2.2892	8.4122±0.9592	0.988	all
Number of H-bonds formed [3]	2.0234±0.9370	2.0875±0.6685	0.589	all

1. Mean and standard deviation (for the 39 structures) of the proportion of residues that satisfy the property shown in the first column.

2. Four residues from the beginning and four from the end of the CDR. 3. In the last two properties, per-residue averages were used instead.

PYMOL COMMAND FOR PDB 5TEZ

```
fetch 5TEZ;
set seq_view, 1;
bg_color white;
hide all;
remove waters;
select beta, chain J and not solvent;
select alpha, chain I and not solvent;
select mhc, (chain A or chain B or chain D or chain E) and not
   solvent;
show cartoon, alpha;
color wheat, alpha;
show cartoon, beta;
color lightblue, beta;
show cartoon,mhc
color grey90, mhc;
create obj_mhc, mhc
show surface, obj_mhc
set transparency=0.2
sel beta_cdr3, (chain J and resi 91:104);
#set cartoon_side_chain_helper, on
#show sticks, beta_cdr3;
#util.cbag beta_cdr3;
color palecyan, beta_cdr3
sel alpha_cdr3, (chain I and resi 91:105);
#set cartoon_side_chain_helper, on
#show sticks, alpha_cdr3;
#util.cbag alpha_cdr3;
color lightpink, alpha_cdr3
select epitope, chain C and not solvent;
show sticks, epitope;
color yellow, epitope
#util.cbay epitope;
#select cdr3, alpha_cdr3 or beta_cdr3;
#select tcr, alpha or beta;
#dist H_cdr_p, cdr3, epitope, mode=2;
#hide labels, H_cdr_p;
#color black, H_cdr_p;
#dist H_cdr_tcr, cdr3, tcr, mode=2;
#hide labels, H_cdr_tcr;
#color grey, H_cdr_tcr;
sel atten_a_head1, (resi 104 and chain I);
#color pink, atten_a_head1;
sel atten_a_head2, (resi 101 and chain I);
#color pink, atten_a_head2;
```

```
sel atten_a_head3, (resi 101 and chain I);
#color pink, atten_a_head3;
sel atten_b_head0, (resi 99 and chain J);
#color pink, atten_b_head0;
show sticks, atten_a_head1
show sticks, atten_a_head2
show sticks, atten_a_head3
show sticks, atten_b_head0
color magenta, atten_a_head1
color magenta, atten_a_head2
color magenta, atten_a_head3
color cyan, atten_b_head0
sel atten_1_int, (resi 93 and chain I)
sel atten_23_int, (resi 94 and chain I)
sel atten_230_int, (resi 6 and chain C)
show sticks, atten_1_int
show sticks, atten_23_int
show sticks, atten_0_int
sel int_int,(resi 98 and chain J)
show sticks, int_int
color atomic,(not elem C)
color gray90, obj_mhc
dist a1_hb,(resi 104 and chain I),(resi 93 and chain I),mode=2
dist a23_hb,(resi 101 and chain I),(resi 94 and chain I),mode=2
dist a23p_hb,(resi 101 and chain I),(resi 6 and chain C),mode=2
dist a0_hb,(resi 99 and chain J),(resi 6 and chain C),mode=2
dist intint,(resi 94 and chain I),(resi 98 and chain J),mode=2
hide labels, a1_hb
hide labels, a23_hb
hide labels, a23p_hb
hide labels, a0_hb
hide labels, intint
```
